# Supplementary material for: Mechanisms of cilia regeneration in Xenopus multiciliated epithelium in vivo
Source: EMBO Rep. 2025 Mar 14;26(8):2192–220. doi: 10.1038/s44319-025-00414-8 (PMC12019409; doi:10.1038/s44319-025-00414-8)

Table of contents

Items	Page numbers
1. List of primers used in this study for cloning Appendix Table S1.	2
2. Legend and Appendix Figure S1.	3-4
3. Legend and Appendix Figure S2.	5-6

Appendix Table S1: Primers were synthesized from IDT for cloning.

ChibbyFwd	ATTCAAGGCCTCTCGAGCCTCTAGAGCCACCATGCCCTTATTCGGGAAC
ChibbyRev	CACCATGGATCCAGATCCTTTCCTCCTGCCAGTCAG
BFPFwd	CAGGAGGAAAGGATCTGGATCCATGGTGTCTAAGGGCGAAG
BFPRev	CTTATCATGTCTGGATCTACGTACTAATTAAGCTTGTGCCCC
SNTNFwd	AATTATGAAAGGATCTGGATCCATGGTGAGCAAGGGCGAG
SNTNRev	GGATCCAGATCCTTTCATAATTTTTACATTCCGTATATTTTGTAGCAGATCTGAC

Appendix Figure S1: Quantification of cilia length and number on CHX and CHX+MG132 treatment.

A) Graph depicting the length of regenerating cilia in the vehicle, CHX, and CHX+MG132 treatments. The cilia lengths at every time point, treatment, and the ‘n’ values are shown in Fig 5D.
B) The Table shows the average cilia length in 3 treatments and 4 time points. Values are represented as mean±SD. The “n” values are shown in Fig. 5D.
C) Table showing variation in cilia number in 3 treatments and 3 time points. Values are represented as mean±SD. The n values and statistical differences are shown in Fig. 5E. ND—Not determined.

Appendix Figure S1

A Quantification of cilia length upon CHX and MG132 treatment

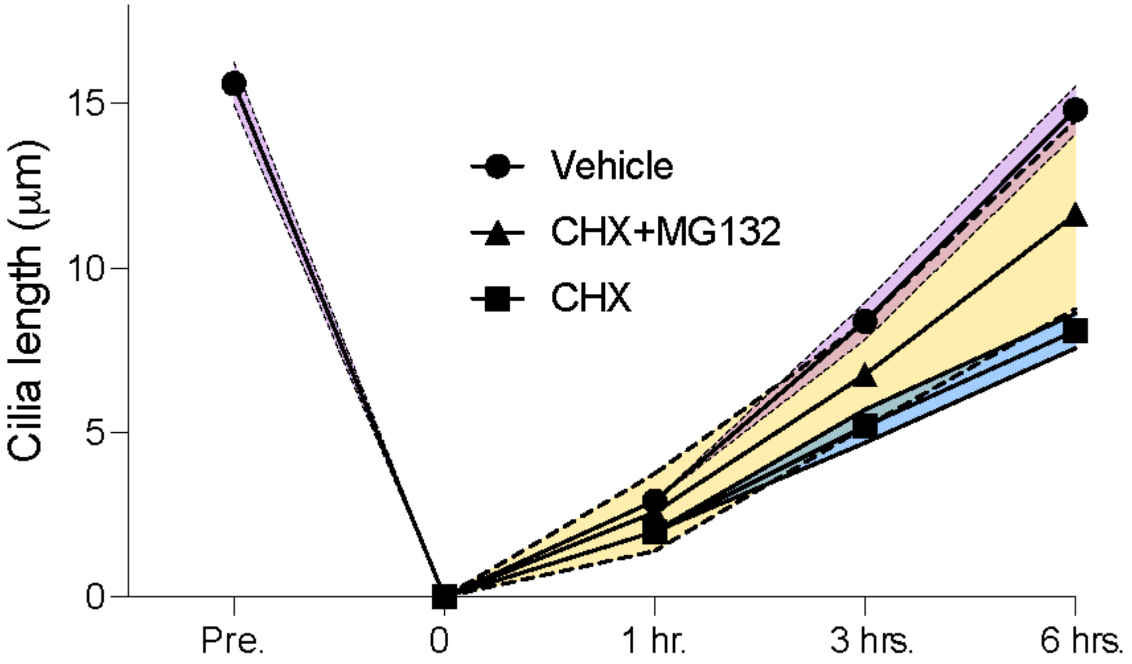

B

	Cilia length in μm		
	**Vehicle**	**CHX**	**CHX+ MG132**
**Pre**	15.61 ± 2.29	ND	ND
**1 hr.**	2.746 ± 1.045	2.242 ± 1.118	2.836 ± 1.494
**3 hrs.**	8.529 ± 1.736	5.4 ± 1.733	6.846 ± 1.534
**6 hrs.**	14.87 ± 2.379	8.798 ± 2.140	11.93 ± 2.529

C

	Cilia number		
	Vehicle	CHX	CHX+ MG132
1 hr.	41.61 ± 12.71	33.78 ± 17.27	31.65 ± 16.31
3 hrs.	ND	14.29 ± 6.164	15.85 ± 5.729
6 hrs.	ND	8.289 ± 3.986	10.28 ± 6.061

Appendix Figure S2: Blocking new protein synthesis does not affect the basal body during regeneration.

A) Stage 28 embryos labeled Chibby-BFP (green, basal bodies) were transferred to DMSO containing 1/9 MR (vehicle) or cycloheximide (CHX) containing 1/9 MR post-deciliation. The embryos were allowed to regenerate and collected at 3 and 6 hrs. Embryos were fixed and stained for cilia (magenta) using Ac. α-Tubulin.
B) The Chibby puncta were counted in all samples (deciliation and regeneration), and the quantification of the basal body number is shown in the graph. The values in parenthesis indicate the number of cells counted from 8 embryos from three trials. ns - not significant, Kruskal-Wallis test, followed by Dunn's test.

Appendix Figure S2

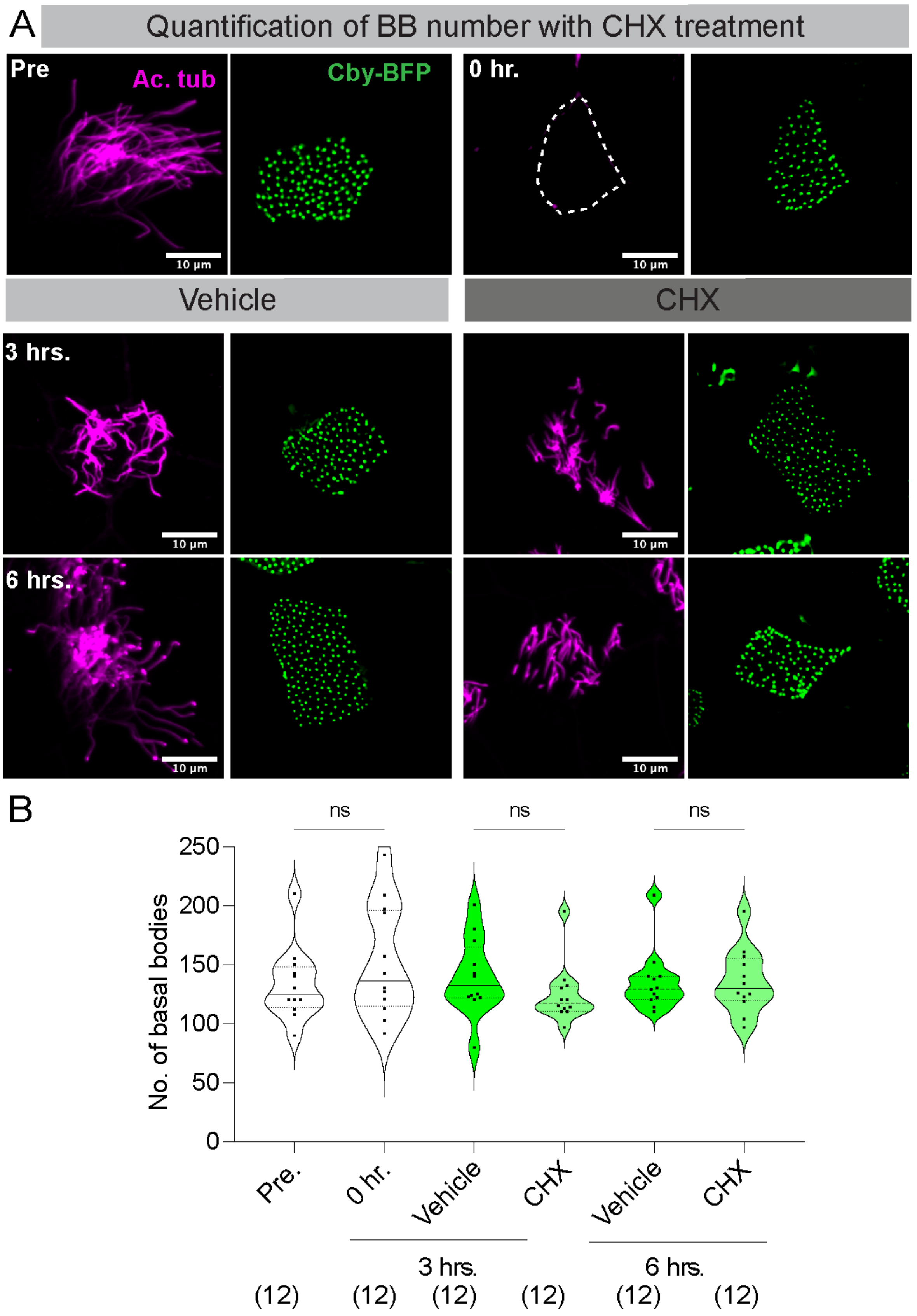

Supplement: Supplementary file 1 — Appendix [file 44319_2025_414_MOESM1_ESM.pdf]
