# Supplementary material for: Mechanisms of cilia regeneration in Xenopus multiciliated epithelium in vivo
Source: EMBO Rep. 2025 Mar 14;26(8):2192–220. doi: 10.1038/s44319-025-00414-8 (PMC12019409; doi:10.1038/s44319-025-00414-8)
Supplement: Supplementary file 4 — Movie EV1 [file 44319_2025_414_MOESM4_ESM.zip › Movie EV 1/Movie EV 1.rtf]

Movie EV1: Mucociliary epithelium regenerates cilia in the same MCC. Live imaging of animal caps dissected from embryos injected with membrane-RFP RNA. Imaging was started approximately 2 minutes post-deciliation. The time stamp on the video is when the imaging was started and not the time of deciliation.-
