# Supplementary material for: Mechanisms of cilia regeneration in Xenopus multiciliated epithelium in vivo
Source: EMBO Rep. 2025 Mar 14;26(8):2192–220. doi: 10.1038/s44319-025-00414-8 (PMC12019409; doi:10.1038/s44319-025-00414-8)
Supplement: Supplementary file 5 — Movie EV2 [file 44319_2025_414_MOESM5_ESM.zip › Movie EV 2/Movie EV 2.rtf]

Movie EV2: Tomograms of Pre deciliated MCC.Cilium from the control sample shows the presence of TZ, indicated by an 'H' shaped electron-dense structure. 
