# Supplementary material for: Mechanisms of cilia regeneration in Xenopus multiciliated epithelium in vivo
Source: EMBO Rep. 2025 Mar 14;26(8):2192–220. doi: 10.1038/s44319-025-00414-8 (PMC12019409; doi:10.1038/s44319-025-00414-8)
Supplement: Supplementary file 7 — Movie EV4 [file 44319_2025_414_MOESM7_ESM.zip › Movie EV 4/Movie EV 4.rtf]

Movie EV4: Tomograms of cilia 20 mins post deciliation.Some cilia have visible basal bodies, but they have not started regenerating 20 minutes after deciliation. They are missing the axoneme and the TZ structure. 
