# Supplementary material for: Mechanisms of cilia regeneration in Xenopus multiciliated epithelium in vivo
Source: EMBO Rep. 2025 Mar 14;26(8):2192–220. doi: 10.1038/s44319-025-00414-8 (PMC12019409; doi:10.1038/s44319-025-00414-8)
Supplement: Supplementary file 8 — Movie EV5 [file 44319_2025_414_MOESM8_ESM.zip › Movie EV 5/Movie EV 5.rtf]

Movie EV5: Tomograms of cilia 20 mins post deciliation.Cilium with a partially built axoneme but missing the H-shaped (TZ) structure after 20 minutes of cilia regeneration. 
