# Supplementary material for: Mechanisms of cilia regeneration in Xenopus multiciliated epithelium in vivo
Source: EMBO Rep. 2025 Mar 14;26(8):2192–220. doi: 10.1038/s44319-025-00414-8 (PMC12019409; doi:10.1038/s44319-025-00414-8)
Supplement: Supplementary file 12 — Movie EV9 [file 44319_2025_414_MOESM12_ESM.zip › Movie EV 9/Movie EV 9.rtf]

Movie EV9: Tomograms of cilia 1 hrs. post deciliation.Cilium with a partially built axoneme with the rudimentary H-shaped (TZ) structure after 1 hr. of cilia regeneration. 
