# Supplementary material for: Mechanisms of cilia regeneration in Xenopus multiciliated epithelium in vivo
Source: EMBO Rep. 2025 Mar 14;26(8):2192–220. doi: 10.1038/s44319-025-00414-8 (PMC12019409; doi:10.1038/s44319-025-00414-8)
Supplement: Supplementary file 14 — Movie EV11 [file 44319_2025_414_MOESM14_ESM.zip › Movie EV 11/Movie EV 11.rtf]

Movie EV11: Tomograms of cilia 1 hrs. post deciliation.Few cilia have visible basal bodies, but they have not started regenerating 1 min after deciliation. They are missing the axoneme and the TZ structure. 
