# Supplementary material for: Mechanisms of cilia regeneration in Xenopus multiciliated epithelium in vivo
Source: EMBO Rep. 2025 Mar 14;26(8):2192–220. doi: 10.1038/s44319-025-00414-8 (PMC12019409; doi:10.1038/s44319-025-00414-8)
Supplement: Supplementary file 15 — Movie EV12 [file 44319_2025_414_MOESM15_ESM.zip › Movie EV 12/Movie EV 12.rtf]

Movie EV12: Tomograms of cilia 1 hrs. post deciliation.TZ structure is missing in samples after 1 hrs. of cilia regeneration.  The axoneme is visible in this tomogram.
