# Supplementary material for: Mechanisms of cilia regeneration in Xenopus multiciliated epithelium in vivo
Source: EMBO Rep. 2025 Mar 14;26(8):2192–220. doi: 10.1038/s44319-025-00414-8 (PMC12019409; doi:10.1038/s44319-025-00414-8)
Supplement: Supplementary file 16 — Movie EV13 [file 44319_2025_414_MOESM16_ESM.zip › Movie EV 13/Movie EV 13.rtf]

Movie EV13: Tomograms of cilia 3 hrs post deciliation.TZ structure is visible and appears near complete in samples after 3 hrs. of cilia regeneration. 
