# Supplementary material for: Mechanisms of cilia regeneration in Xenopus multiciliated epithelium in vivo
Source: EMBO Rep. 2025 Mar 14;26(8):2192–220. doi: 10.1038/s44319-025-00414-8 (PMC12019409; doi:10.1038/s44319-025-00414-8)
Supplement: Supplementary file 17 — Movie EV14 [file 44319_2025_414_MOESM17_ESM.zip › Movie EV 14/Movie 14.rtf]

Movie EV14: Tomograms of cilia 6 hrs. post deciliation.TZ structure is visible and appears similar to controls in samples after 6 hrs. of cilia regeneration. 
