# Supplementary material for: Mechanisms of cilia regeneration in Xenopus multiciliated epithelium in vivo
Source: EMBO Rep. 2025 Mar 14;26(8):2192–220. doi: 10.1038/s44319-025-00414-8 (PMC12019409; doi:10.1038/s44319-025-00414-8)
Supplement: Supplementary file 19 — Source data Fig. 1 [file 44319_2025_414_MOESM19_ESM.zip › Figure 1/Read me_1A.rtf]

Figure 1 1B. Each folder has subfolder that contains uncropped unmodified images of  Whole embryo (WE) tiled image with 2 channels Actin and Acetylated tubulin (labelled as WE_timepoint_AC tub or WE_timepoint_Actin) A Zoomed in image of the Ac tubulin channel (labelled as Zoom_timepoint_AC tub) SEM image(labelled as SEM_timepoint). Time points- Pre., 0 hr., 1 hr., 3 hrs., 6 hrs.
