# Supplementary material for: Mechanisms of cilia regeneration in Xenopus multiciliated epithelium in vivo
Source: EMBO Rep. 2025 Mar 14;26(8):2192–220. doi: 10.1038/s44319-025-00414-8 (PMC12019409; doi:10.1038/s44319-025-00414-8)
Supplement: Supplementary file 20 — Source data Fig. 2 [file 44319_2025_414_MOESM20_ESM.zip › Figure 2/Read me_2 A and B.rtf]

Figure 2 2A.  Each folder has subfolders that contains uncropped unmodified images (TIFF) of Ac tub and B9D1 channels labelled as (Timepoint_Ac tub or Timepoint_B9D1)Time points- Pre., 0 hr., 1 hr., 3 hrs.For final figure the brightness contrast was adjusted and cropped around each cell in Fiji, scale bar was added and saved as tiff.  2B.    Each folder contains uncropped unmodified TEM images labelled as per timepoints. Time points Pre., 0 hr., 20 mins., 90 mins. For the final figure, the images were cropped around the ROI in Fiji and saved as Tiff. 
