# Supplementary figures and images for: Mechanisms of cilia regeneration in Xenopus multiciliated epithelium in vivo
Source: EMBO Rep. 2025 Mar 14;26(8):2192–220. doi: 10.1038/s44319-025-00414-8 (PMC12019409; doi:10.1038/s44319-025-00414-8)

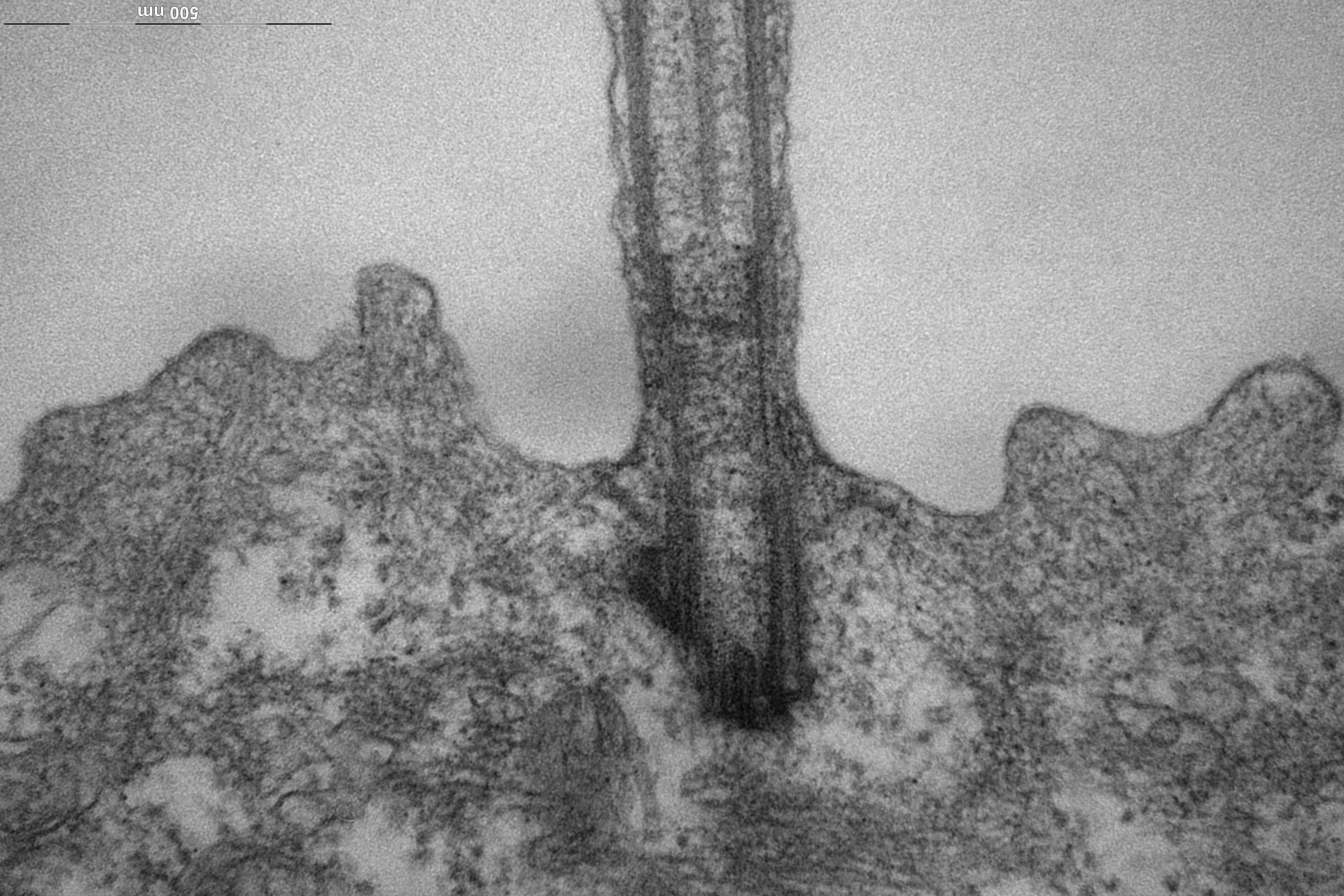

Supplement: Supplementary file 20 — Source data Fig. 2 [file 44319_2025_414_MOESM20_ESM.zip › Figure 2/2B/90 mins.tif]

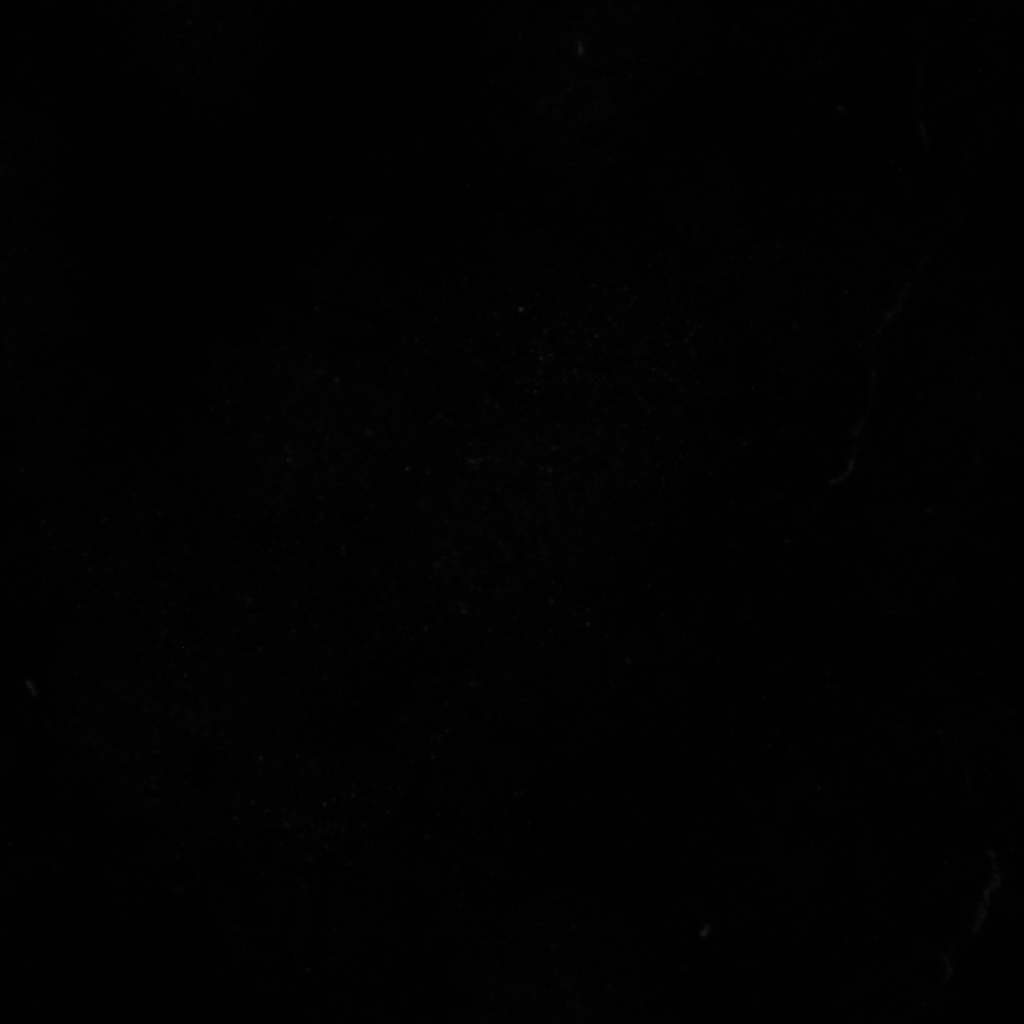

Supplement: Supplementary file 20 — Source data Fig. 2 [file 44319_2025_414_MOESM20_ESM.zip › Figure 2/2A/0 hr/0 hr_Ac ub.tif]

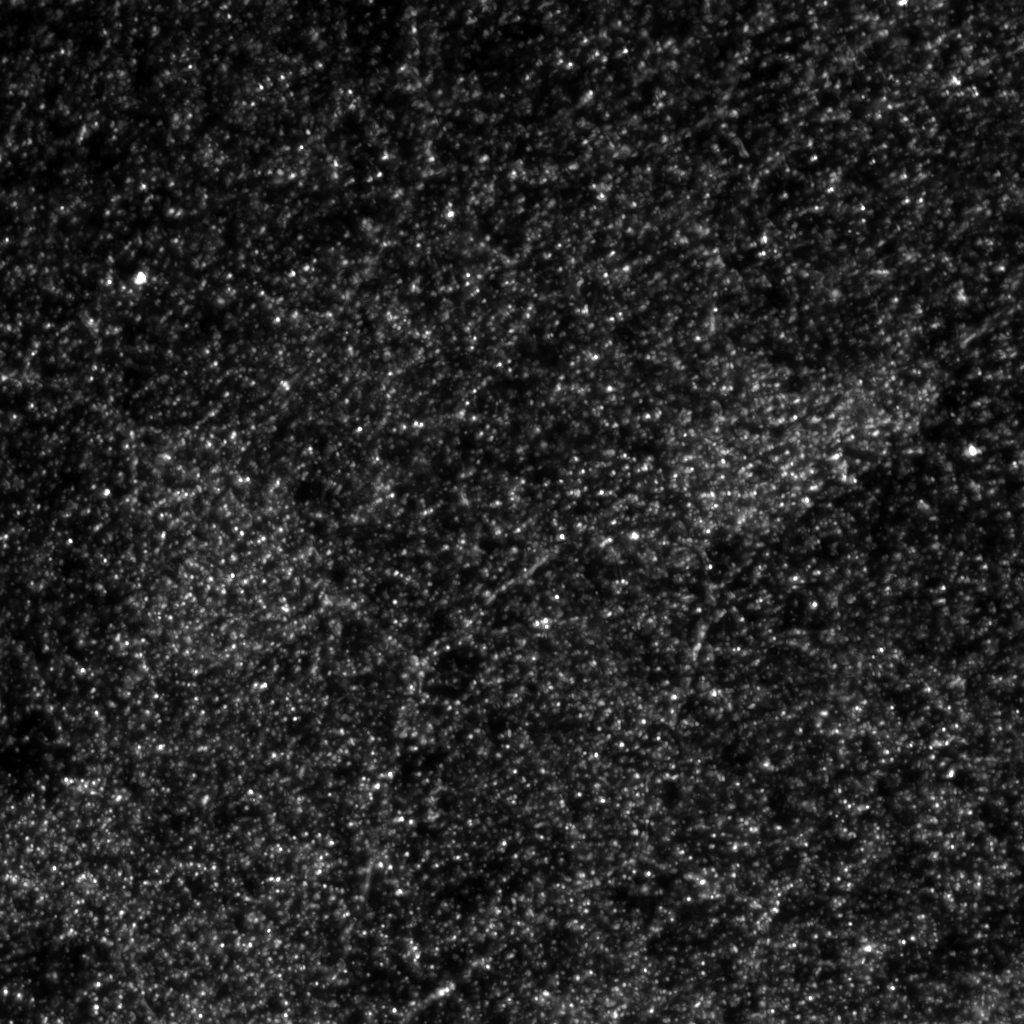

Supplement: Supplementary file 22 — Source data Fig. 4 [file 44319_2025_414_MOESM22_ESM.zip › Figure 4/4A/0 hr/0 hr_B9D1.tif]

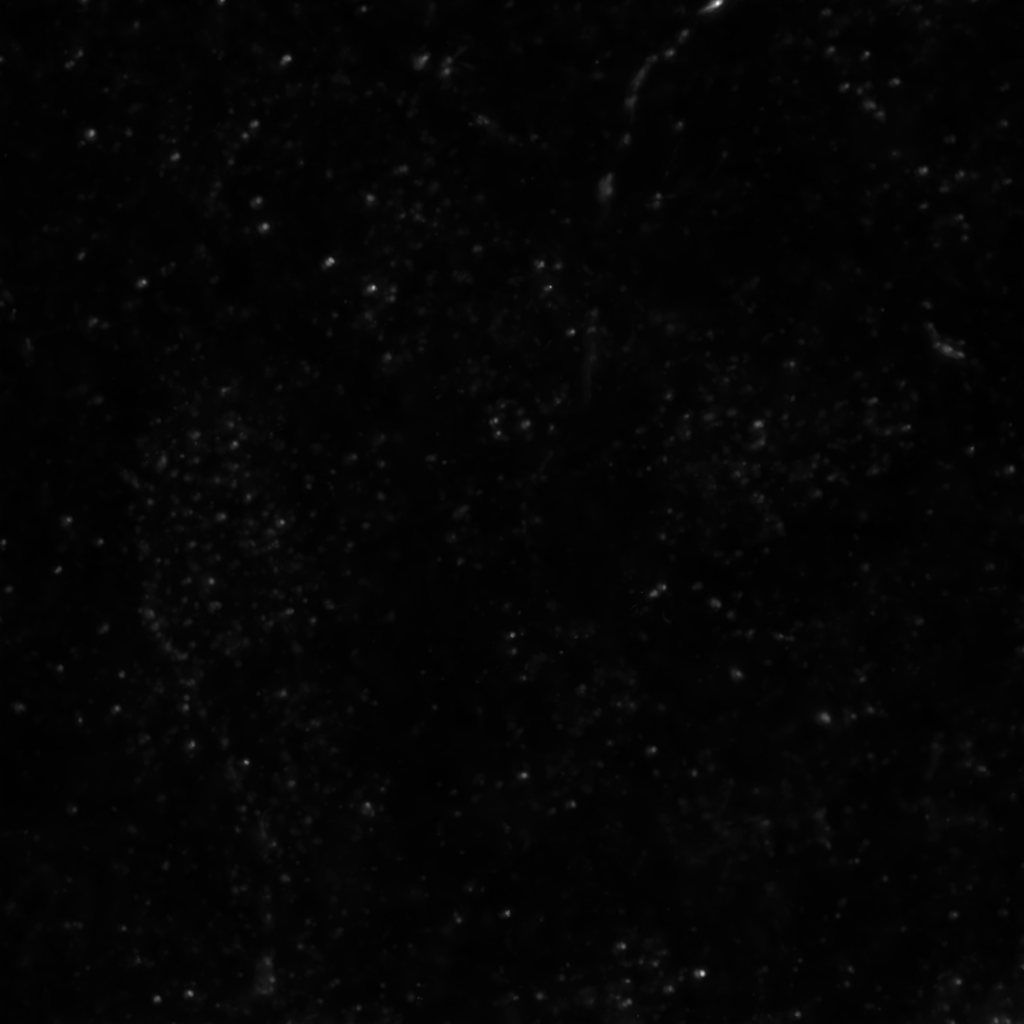

Supplement: Supplementary file 22 — Source data Fig. 4 [file 44319_2025_414_MOESM22_ESM.zip › Figure 4/4A/0 hr/0 hr_Act Tub.tif]

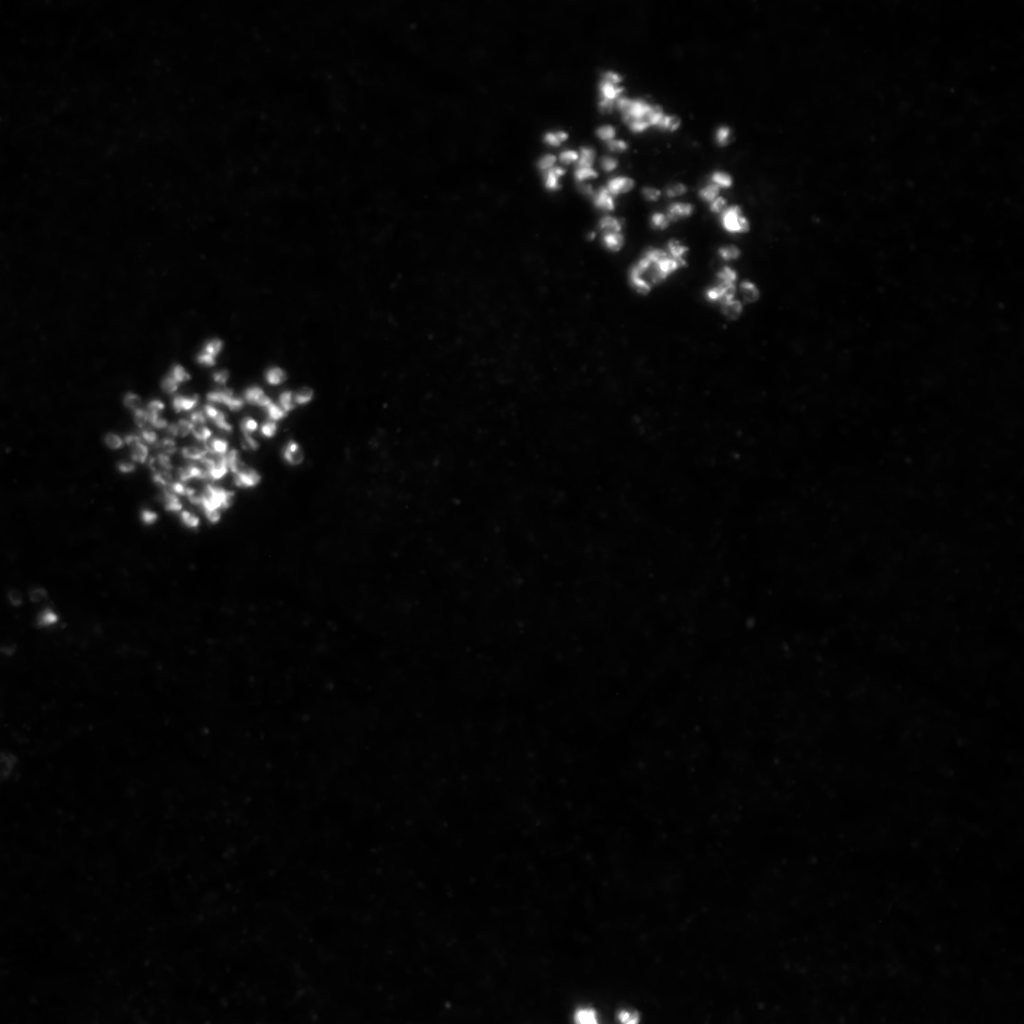

Supplement: Supplementary file 22 — Source data Fig. 4 [file 44319_2025_414_MOESM22_ESM.zip › Figure 4/4A/Control 2 hr/Ctrl_2hr_Act Tub.tif]

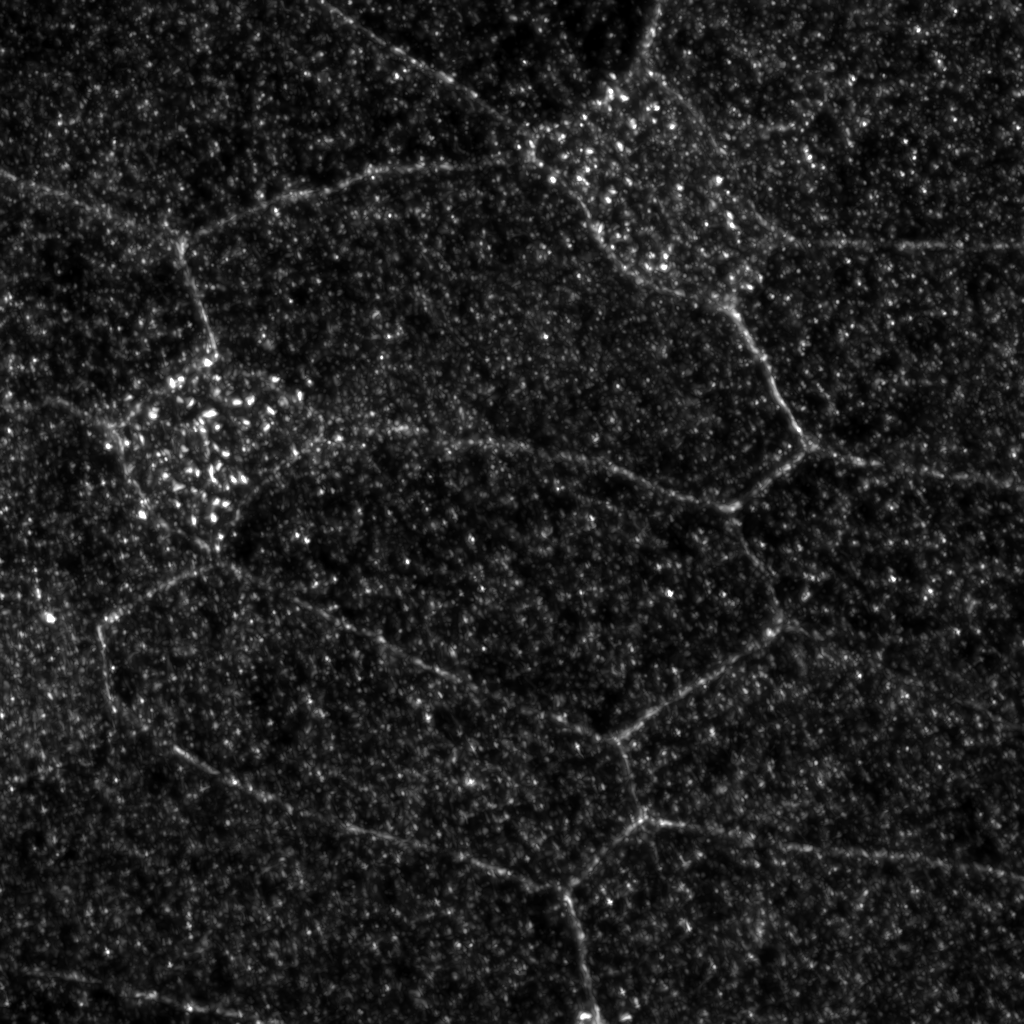

Supplement: Supplementary file 22 — Source data Fig. 4 [file 44319_2025_414_MOESM22_ESM.zip › Figure 4/4A/Control 2 hr/Ctrl_2hr_B9D1.tif]

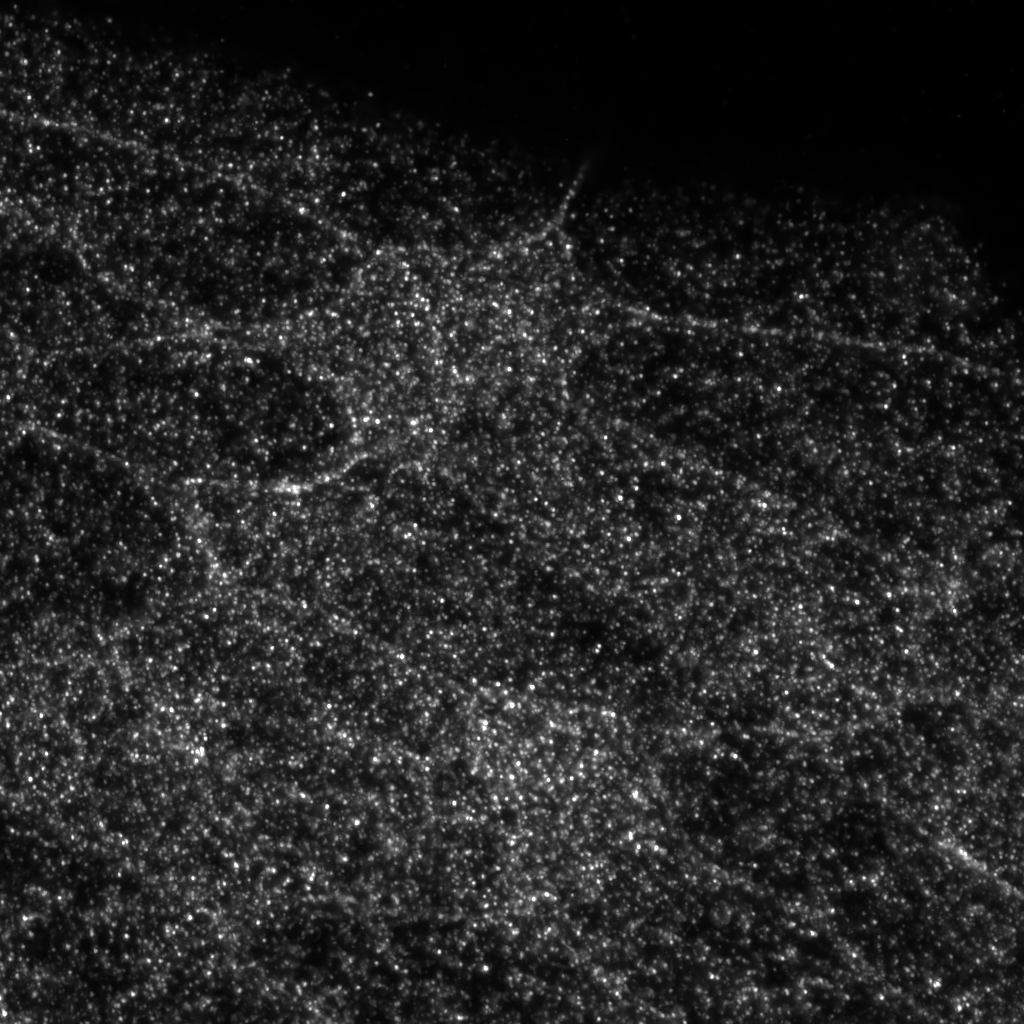

Supplement: Supplementary file 22 — Source data Fig. 4 [file 44319_2025_414_MOESM22_ESM.zip › Figure 4/4A/CHX 2 hr/CHX_2hr_B9D1.tif]

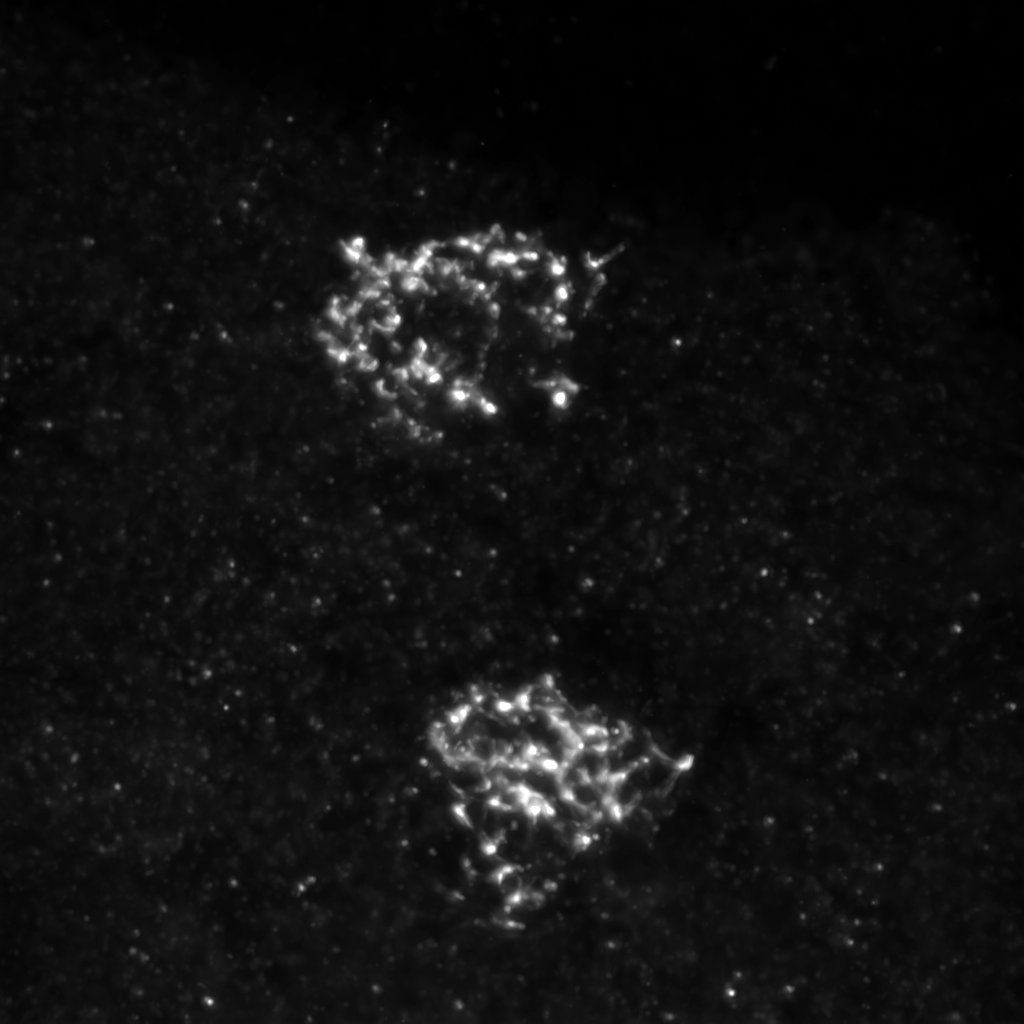

Supplement: Supplementary file 22 — Source data Fig. 4 [file 44319_2025_414_MOESM22_ESM.zip › Figure 4/4A/CHX 2 hr/CHX_2hr_Act Tub.tif]

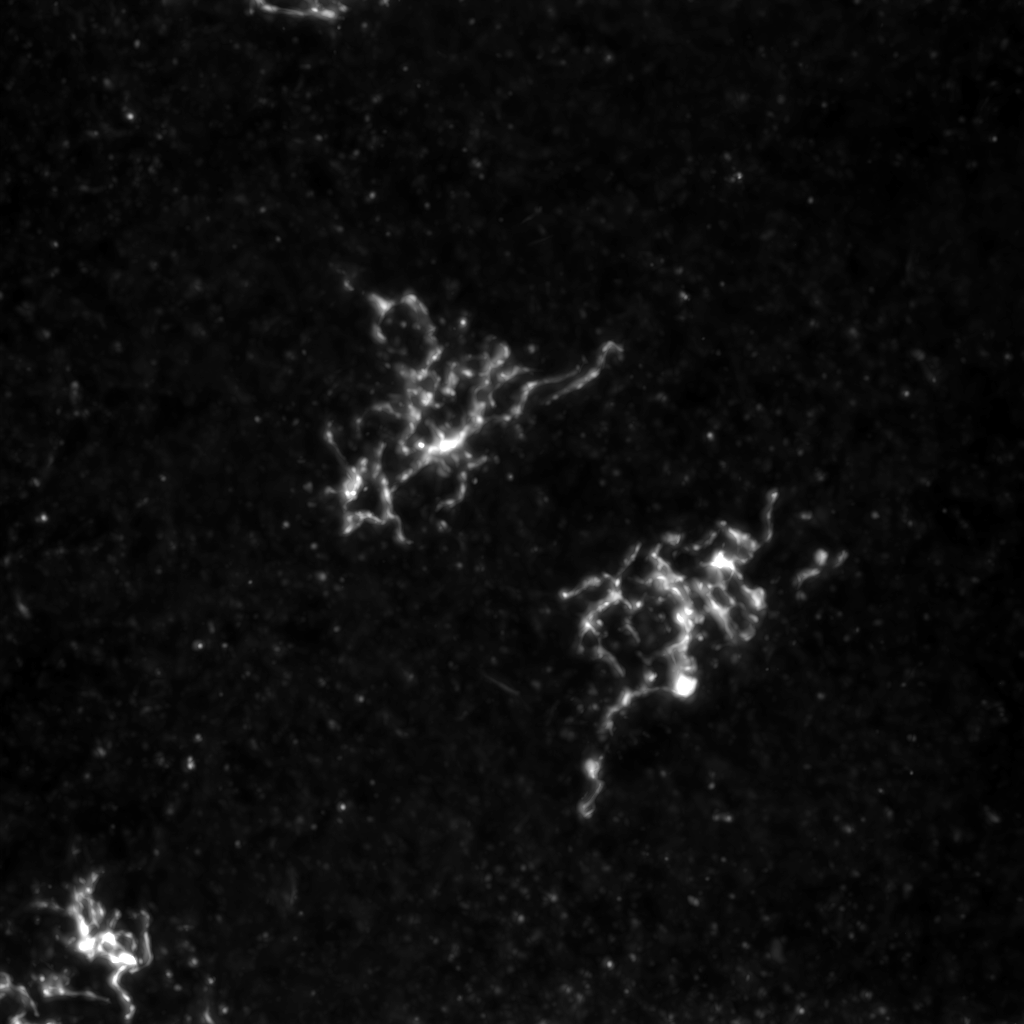

Supplement: Supplementary file 22 — Source data Fig. 4 [file 44319_2025_414_MOESM22_ESM.zip › Figure 4/4A/CHX 3 hr/CHX_3hr_Act Tub.tif]

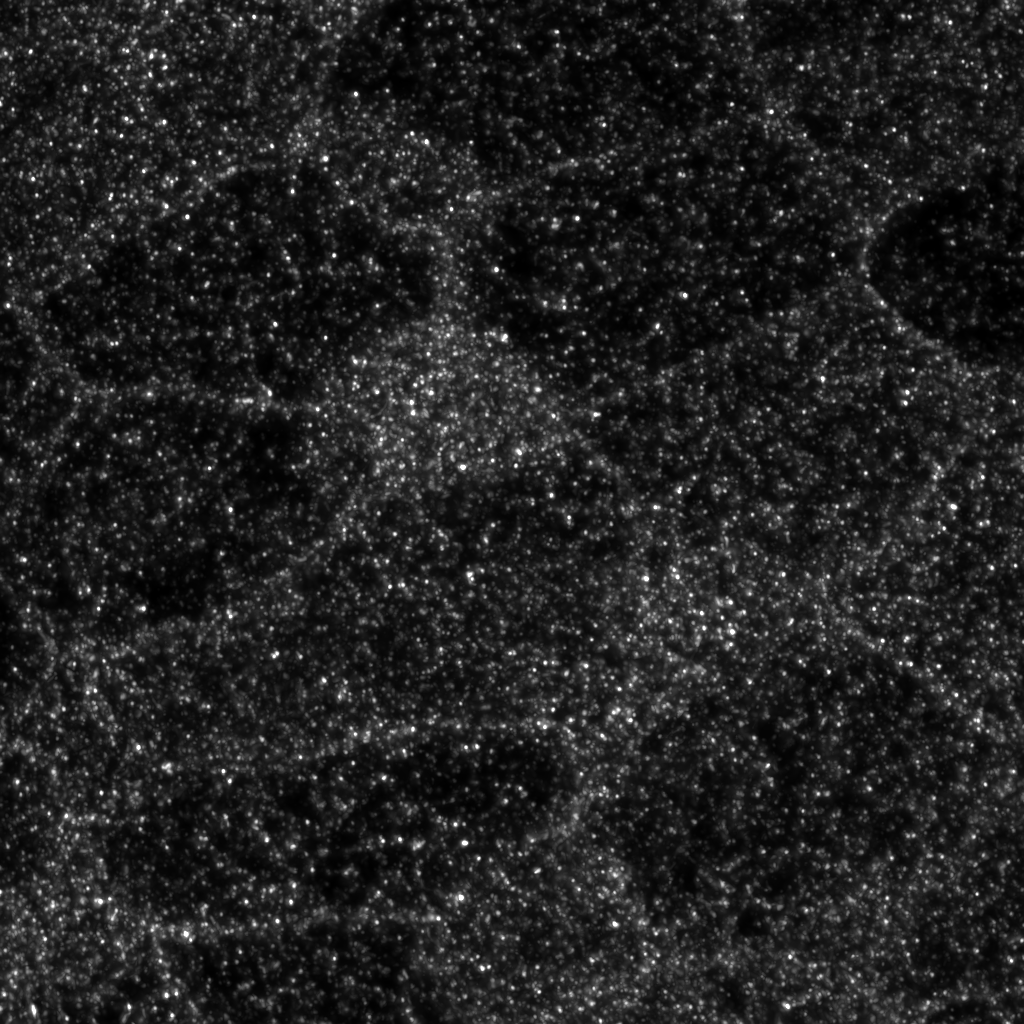

Supplement: Supplementary file 22 — Source data Fig. 4 [file 44319_2025_414_MOESM22_ESM.zip › Figure 4/4A/CHX 3 hr/CHX_3hr_B9D1.tif]

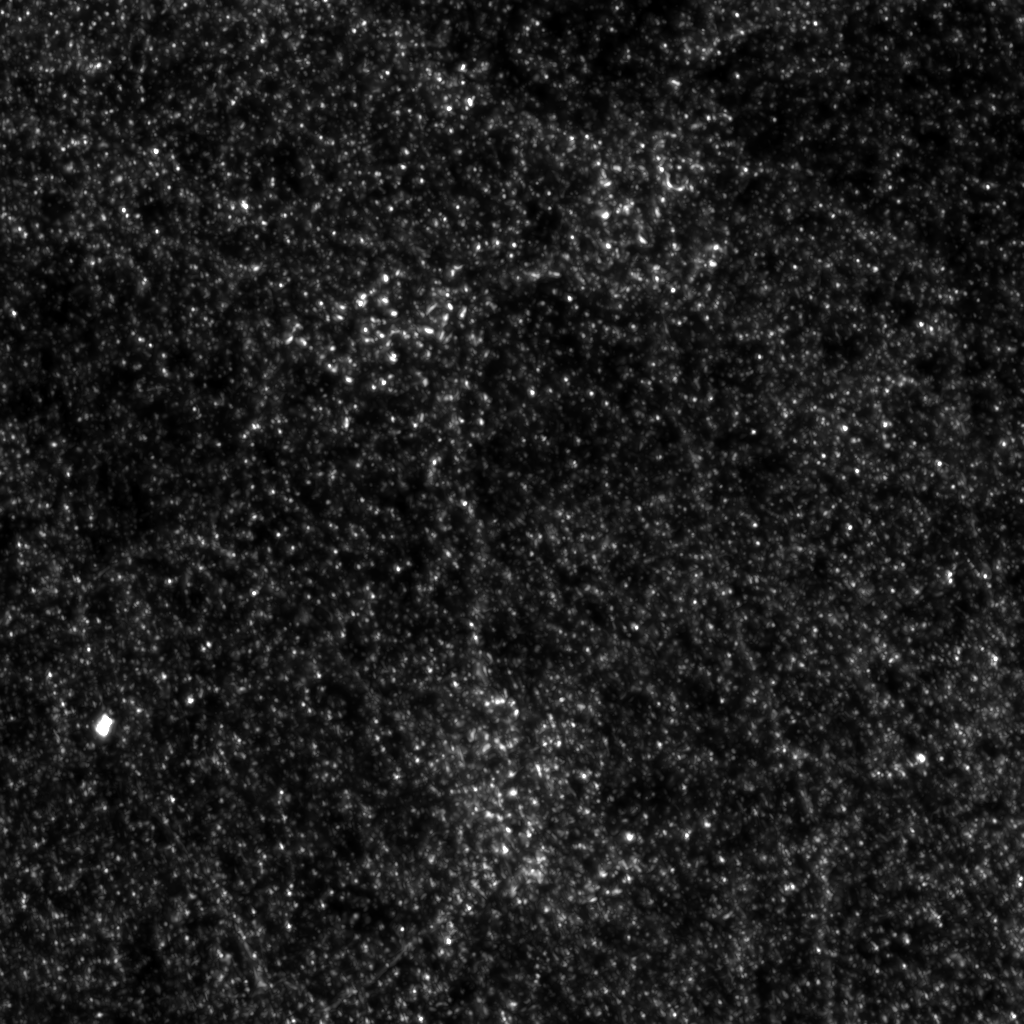

Supplement: Supplementary file 22 — Source data Fig. 4 [file 44319_2025_414_MOESM22_ESM.zip › Figure 4/4A/Control 3 hr/Ctrl_3hr_B9D1.tif]

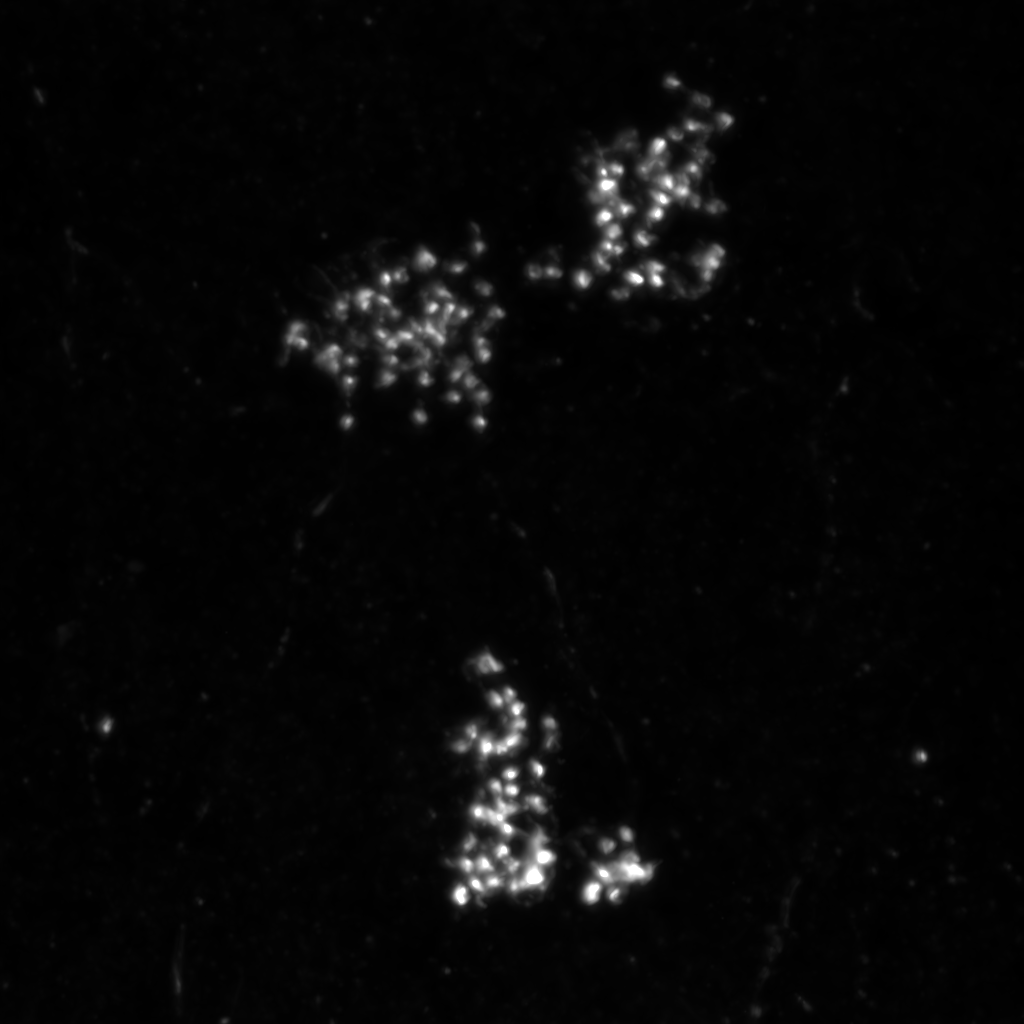

Supplement: Supplementary file 22 — Source data Fig. 4 [file 44319_2025_414_MOESM22_ESM.zip › Figure 4/4A/Control 3 hr/Ctrl_3hr_Act Tub.tif]

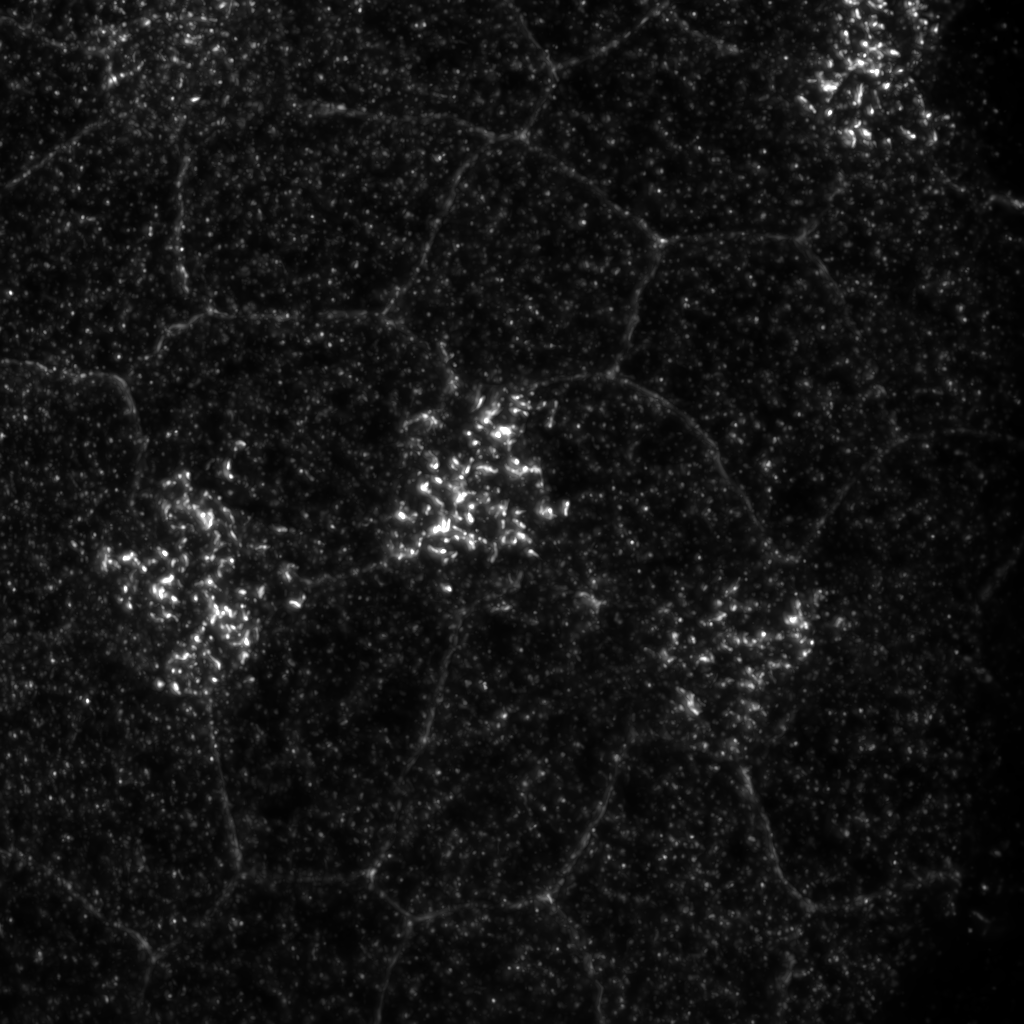

Supplement: Supplementary file 22 — Source data Fig. 4 [file 44319_2025_414_MOESM22_ESM.zip › Figure 4/4A/Pre/Pre_B9D1.tif]

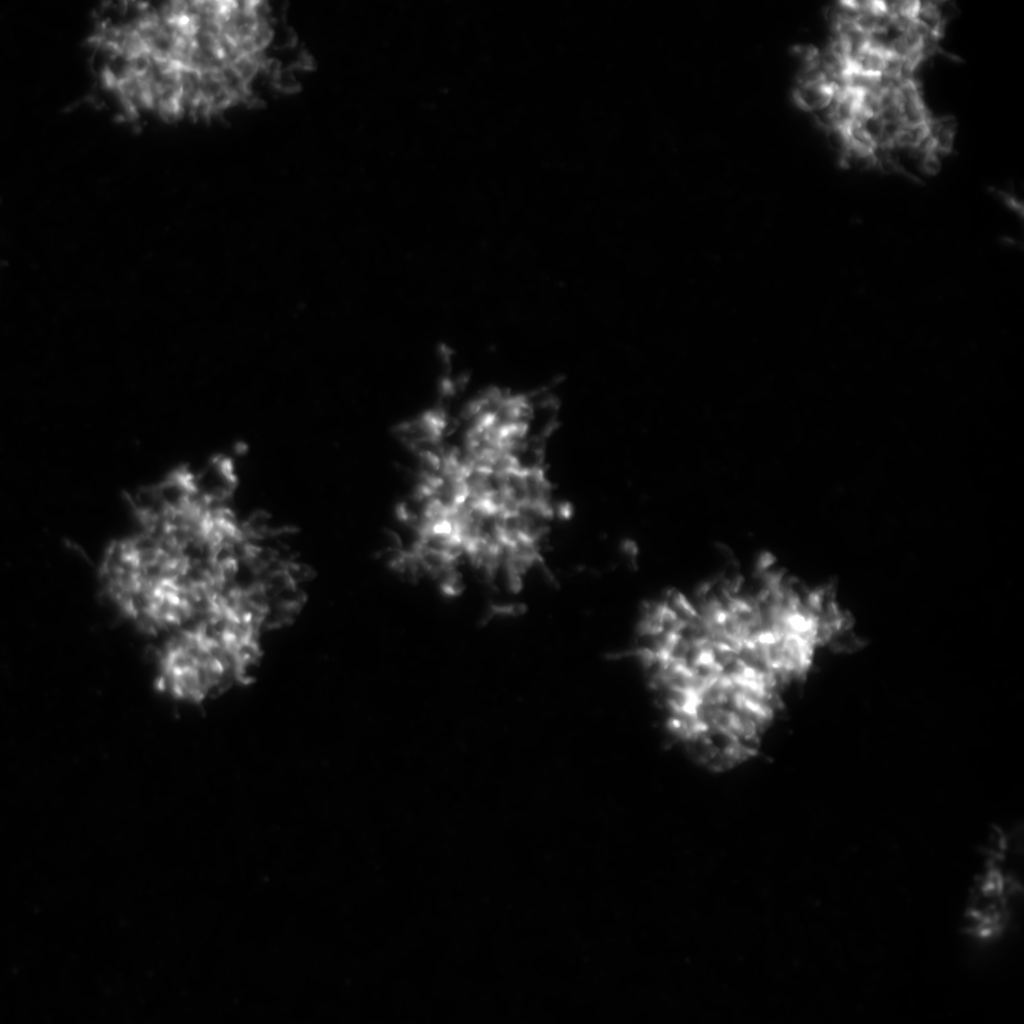

Supplement: Supplementary file 22 — Source data Fig. 4 [file 44319_2025_414_MOESM22_ESM.zip › Figure 4/4A/Pre/Pre_Ac Tub.tif]

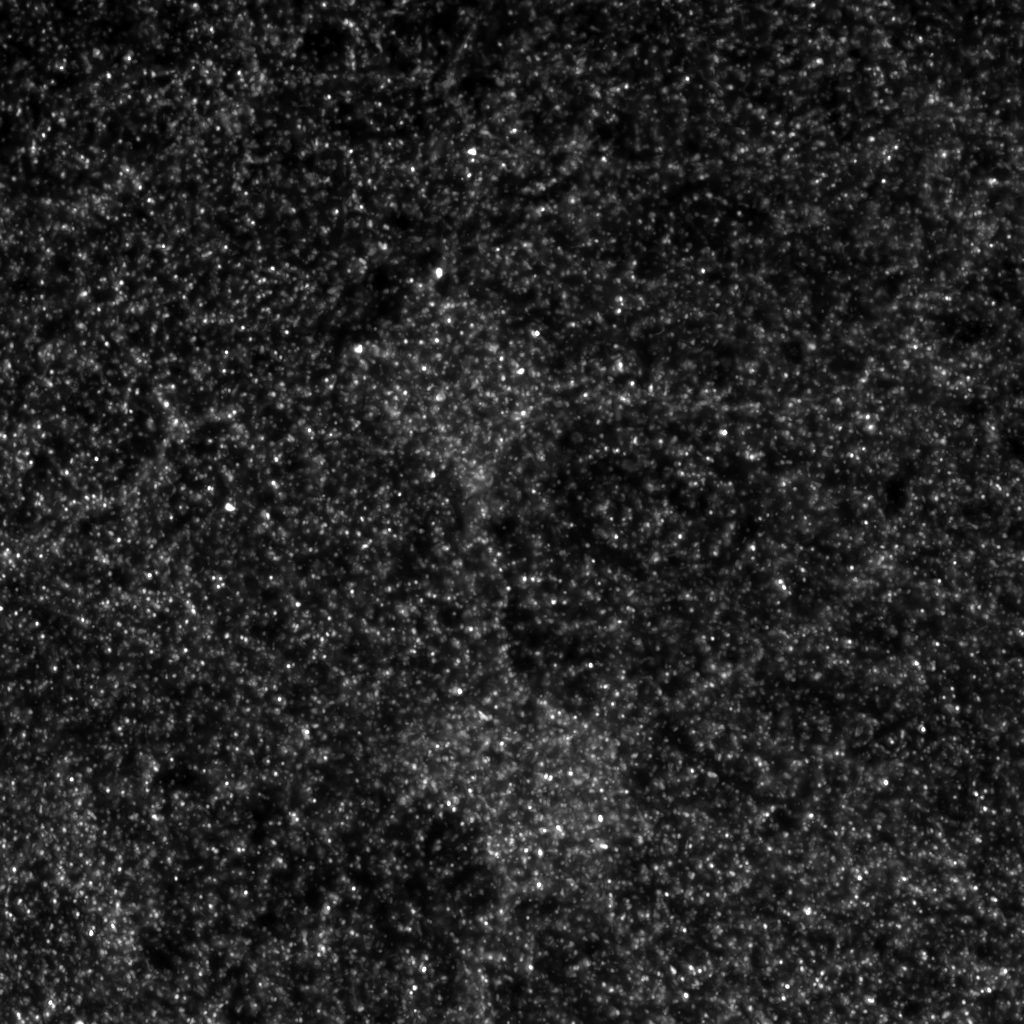

Supplement: Supplementary file 22 — Source data Fig. 4 [file 44319_2025_414_MOESM22_ESM.zip › Figure 4/4A/Control 1 hr/Ctrl_1hr_B9D1.tif]

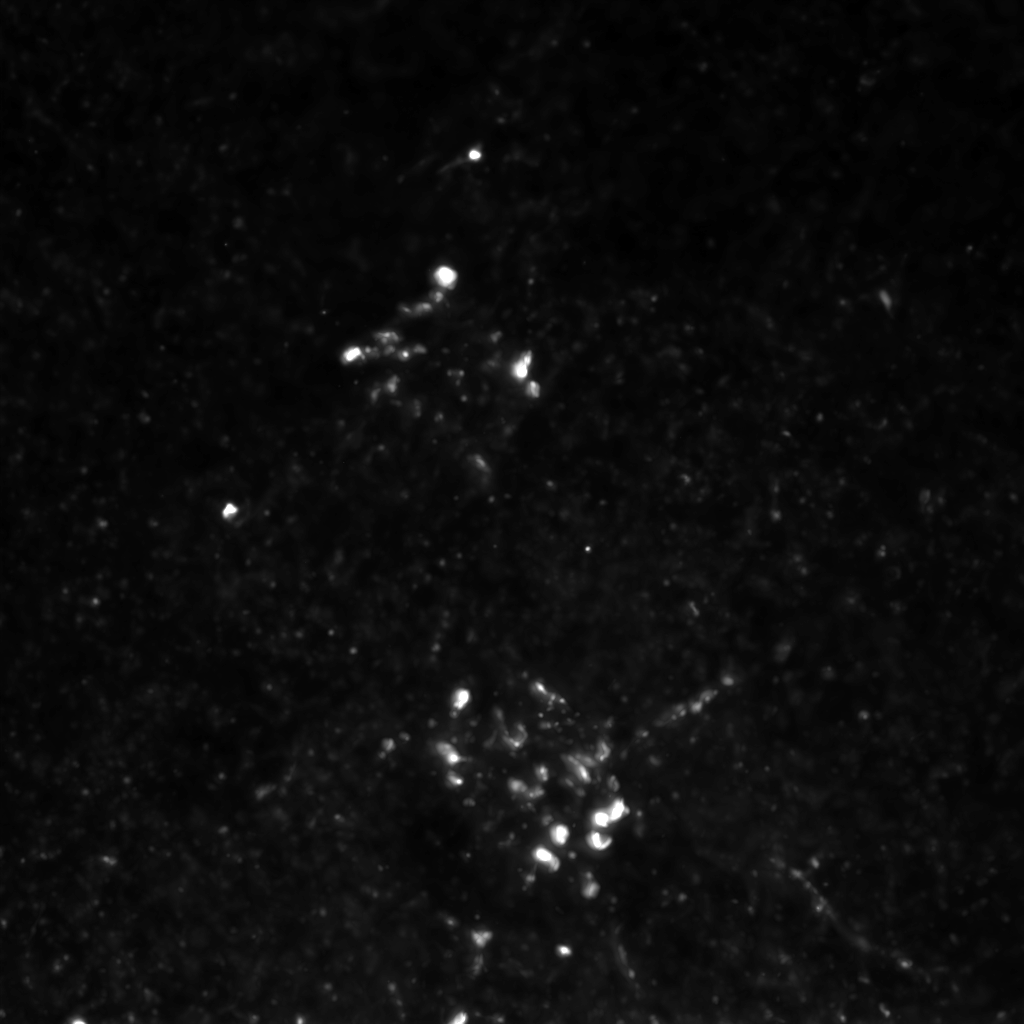

Supplement: Supplementary file 22 — Source data Fig. 4 [file 44319_2025_414_MOESM22_ESM.zip › Figure 4/4A/Control 1 hr/Ctrl 1 hr_Act Tub.tif]

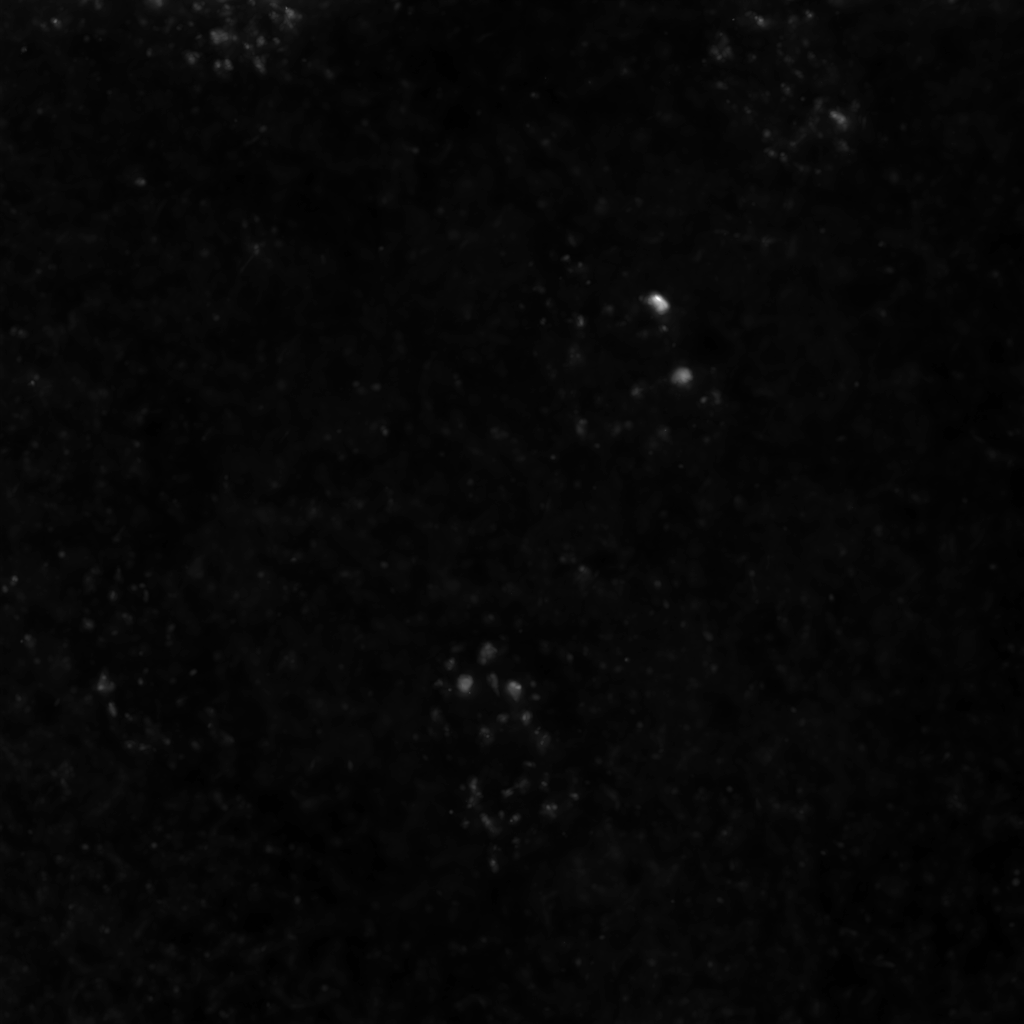

Supplement: Supplementary file 22 — Source data Fig. 4 [file 44319_2025_414_MOESM22_ESM.zip › Figure 4/4A/CHX 1 hr/CHX_1hr_Act Tub.tif]

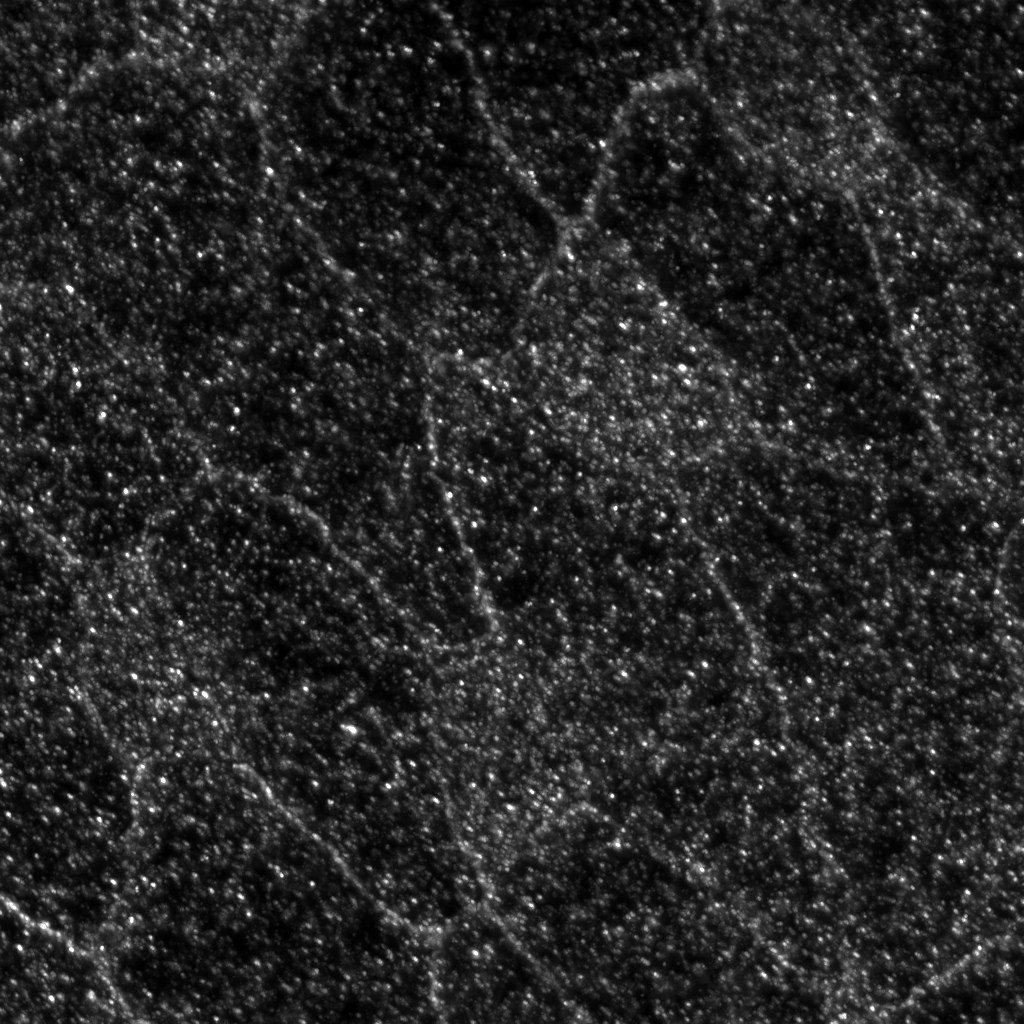

Supplement: Supplementary file 22 — Source data Fig. 4 [file 44319_2025_414_MOESM22_ESM.zip › Figure 4/4A/CHX 1 hr/CHX_1hr_B9D1.tif]
