# Supplementary material for: Mechanisms of cilia regeneration in Xenopus multiciliated epithelium in vivo
Source: EMBO Rep. 2025 Mar 14;26(8):2192–220. doi: 10.1038/s44319-025-00414-8 (PMC12019409; doi:10.1038/s44319-025-00414-8)
Supplement: Supplementary file 21 — Source data Fig. 3 [file 44319_2025_414_MOESM21_ESM.zip › Figure 3/Read me_3A.rtf]

Figure 3 3 A. Folder has sub folders that contains uncropped unmodified images (TIFF) of  memGFP and mScarSNTN channels labelled as (Timepoint_memGFP or Timepoint_mScarSNTN) Time points Pre., 0 hr., 1 hr., 2 hr., 3 hr., 6 hr. For final figure the brightness contrast was adjusted and cropped around each cell in Fiji, scale bar was added and saved as tiff. A dotted outline was drawn around the inset boundary using the Fiji dotted line plugin before saving the images. The inset was cropped from the saved tiff image from each channel. 
