# Supplementary material for: Mechanisms of cilia regeneration in Xenopus multiciliated epithelium in vivo
Source: EMBO Rep. 2025 Mar 14;26(8):2192–220. doi: 10.1038/s44319-025-00414-8 (PMC12019409; doi:10.1038/s44319-025-00414-8)
Supplement: Supplementary file 22 — Source data Fig. 4 [file 44319_2025_414_MOESM22_ESM.zip › Figure 4/Read me_4A.rtf]

Figure 4 4A.  Folder has sub folders that contains uncropped unmodified images (TIFF) of Ac tub and B9D1 channels of control and CHX treated samples labelled as (Ctrl_Timepoint_Ac tub/Ctrl_Timepoint_B9D1 or CHX_Timepoint_Ac tub/CHX_Timepoint_B9D1) at different time points. Time points- Pre., 0 hr., 1 hr.,2hrs., 3 hrs. For final figure the brightness contrast was adjusted and cropped around each cell in Fiji, scale bar was added and saved as tiff. A dotted outline was also drawn around each cell to mark the cell and the inset boundary using the Fiji dotted line plugin before saving the images. The inset was cropped from the saved tiff image from each channel.
