# Supplementary material for: Mechanisms of cilia regeneration in Xenopus multiciliated epithelium in vivo
Source: EMBO Rep. 2025 Mar 14;26(8):2192–220. doi: 10.1038/s44319-025-00414-8 (PMC12019409; doi:10.1038/s44319-025-00414-8)
Supplement: Supplementary file 23 — Source data Fig. 5 [file 44319_2025_414_MOESM23_ESM.zip › Figure 5/Read me_5B and C.rtf]

Figure 5 5B.   Folder has sub folders that contains uncropped unmodified images (TIFF) of Ac tub and Actin channels of Pre deciliated and 0hr timepoints  (labelled as Timepoint_AC tub Timepoint _Actin) Time points Pre., 0 hr,        5C. Folder has sub foders that contains uncropped unmodified images (TIFF) of Ac tub and Actin channels from treated samples (labelled as Treatment_Timepoint_Ac tub/ Treatment_ Timepoint _Actin) at different time points Treatments are Vehicle, CHX, and CHX+MG132, time points are 1 hr, 3 hrs., and 6 hrs.  For final figure the brightness contrast was adjusted and cropped around each cell in Fiji, scale bar was added and saved as tiff.
