# Supplementary material for: Mechanisms of cilia regeneration in Xenopus multiciliated epithelium in vivo
Source: EMBO Rep. 2025 Mar 14;26(8):2192–220. doi: 10.1038/s44319-025-00414-8 (PMC12019409; doi:10.1038/s44319-025-00414-8)
Supplement: Supplementary file 24 — Source data Fig. 7 [file 44319_2025_414_MOESM24_ESM.zip › Figure 7/Read me_7 A_A'_A".rtf]

Figure 7 7A. Folder has subfolder that contains uncropped unmodified images (TIFF) of IFT43, Ac Tub and Centrin channels of Pre deciliated and 0 cells (labelled as Timepoint_Ac tub Timepoint _IFT43, Time point_mRFP Centrin)Time points Pre., 0 hr.       7A`Folder has subfolders that contains uncropped unmodified images (TIFF) of IFT43, Ac Tub and Centrin images of cells treated with vehicle at different time points (labelled as Veh_Timepoint_Ac tub., Veh_Timepoint _IFT43, Veh_Time point_mRFP Centrin). Time points are 1 hr, 3 hrs., and 6 hrs.  7A`` Folder has subfolders that contains uncropped unmodified images (TIFF) of IFT43, Ac Tub and Centrin images of cells treated with CHX (labelled as CHX_Timepoint_AC tub., CHX_Timepoint _IFT43, CHX_Time point_mRFP Centrin) at different time points. Time points are 1 hr., 3 hrs., and 6 hrs.
