# Supplementary material for: Mechanisms of cilia regeneration in Xenopus multiciliated epithelium in vivo
Source: EMBO Rep. 2025 Mar 14;26(8):2192–220. doi: 10.1038/s44319-025-00414-8 (PMC12019409; doi:10.1038/s44319-025-00414-8)
Supplement: Supplementary file 25 — Expanded View Figures [file 44319_2025_414_MOESM25_ESM.pdf]

Expanded View Figures

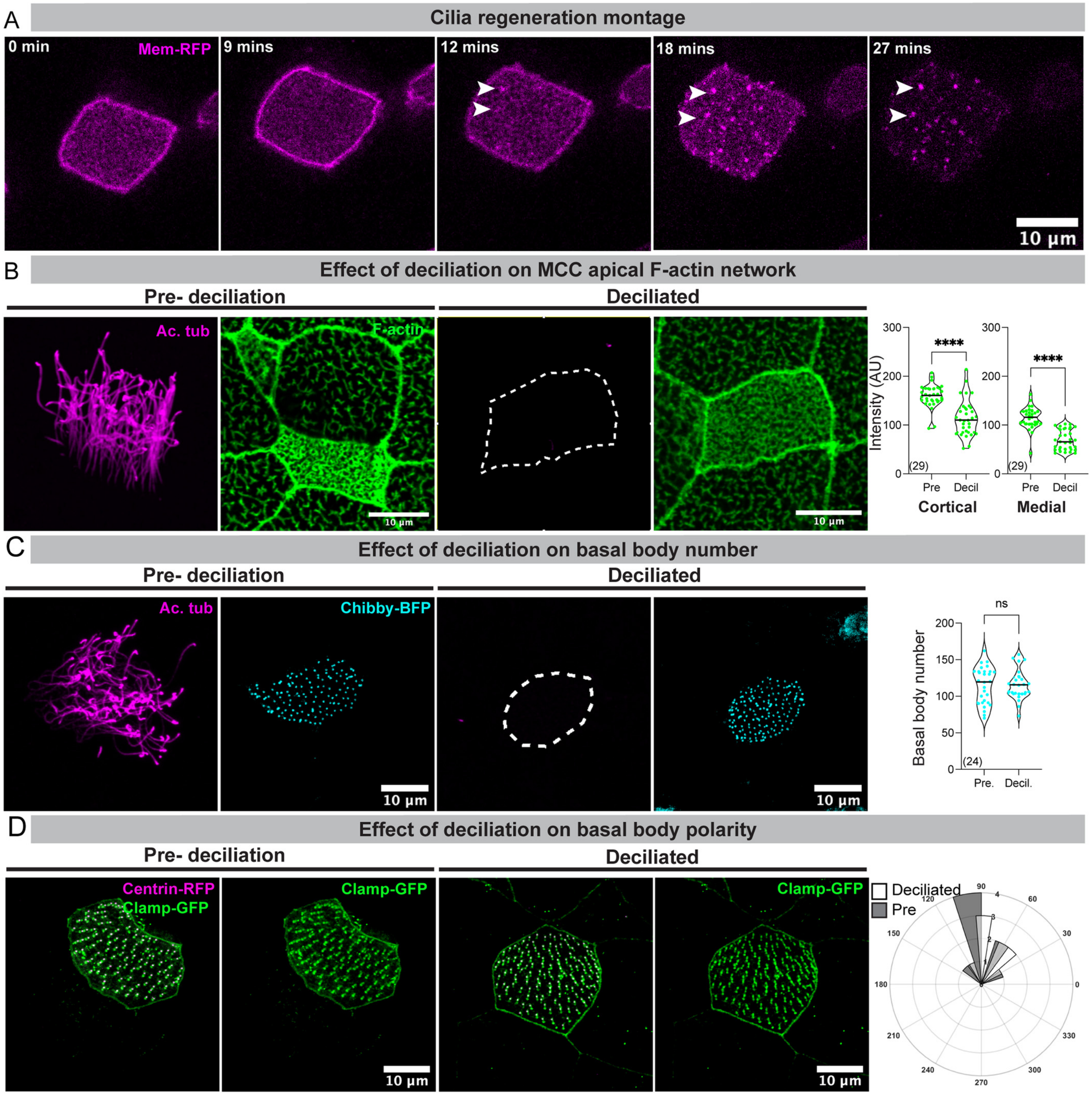

Figure EV1. Deciliation affects apical F-actin, but the basal body number is unaffected.

(**A**) Montage of regenerating cilia (mem-RFP) in the animal caps. The mem-RFP signal specks (marked by white arrows) can be seen emerging from the cell's surface by ~12 min, eventually growing into beating cilia (see Movie EV1). (**B**) Pre- and post-deciliation MCCs that are stained for cilia(magenta) and F-actin(green) are depicted. Cortical and medial F-actin intensity significantly differs in Pre and 0-h deciliated samples. The values in parenthesis indicate the number of MCCs measured from three trials from 9–10 embryos. ****$P < 0.0001$, Mann–Whitney test. (**C**) The number of basal bodies labeled with Chibby-GFP (cyan) is unaffected after deciliation. The values in parenthesis indicate the number of MCCs measured from three trials from 9–10 embryos. ns = not significant, Mann–Whitney test. (**D**) Deciliation does not affect basal body polarity. Basal body polarity was determined by measuring the orientation of rootlets labeled with Clamp-GFP (green) with their respective basal body labeled with Centrin-RFP (magenta) and represented in the rose plot. The directionality was measured in 27 pre-deciliated cells and 22 MCCs post-deciliation from three trials from 9–10 embryos in each category.

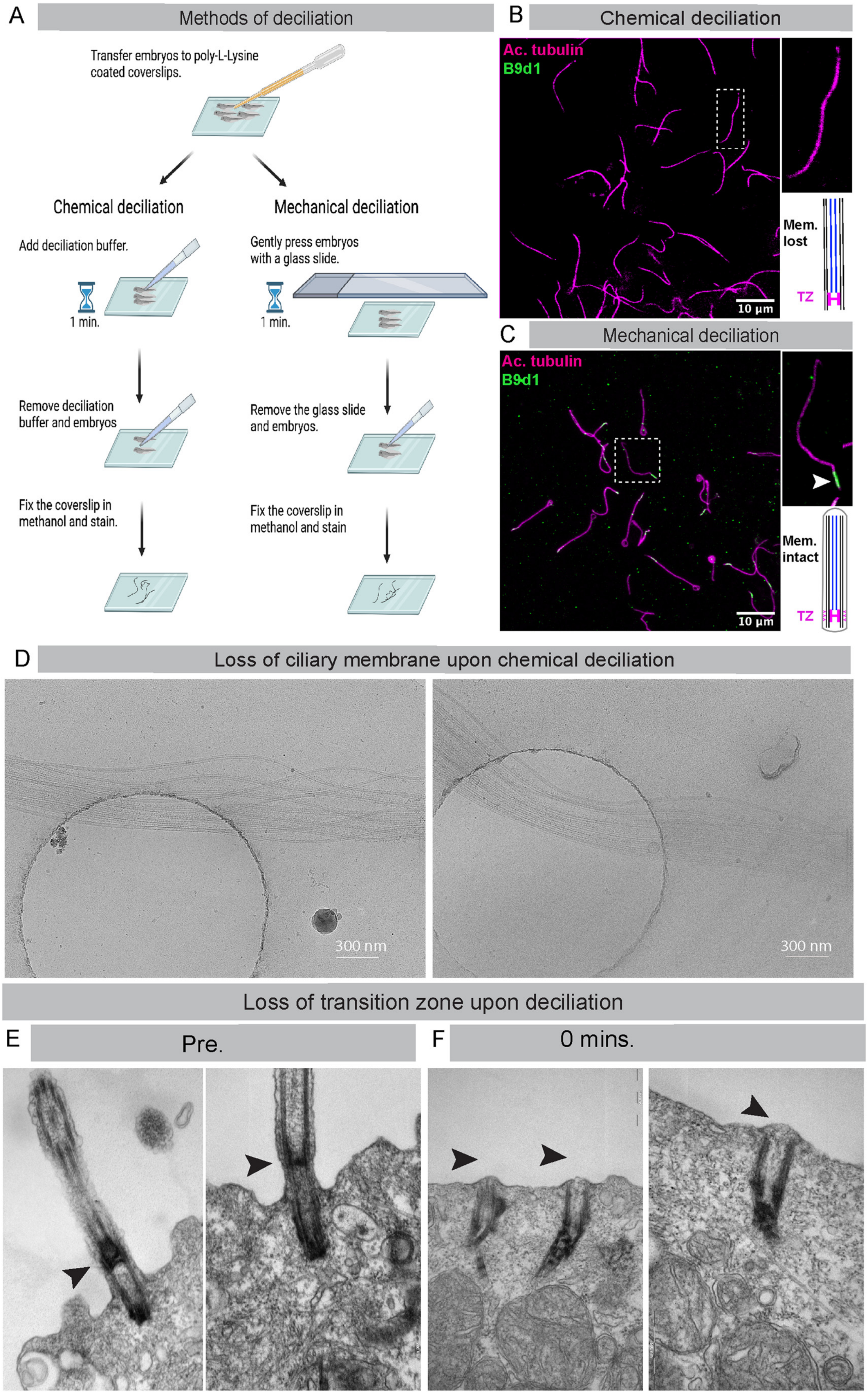
A
Methods of deciliation
Transfer embryos to poly-L-Lysine coated coverslips.
Chemical deciliation
Mechanical deciliation
Add deciliation buffer.
1 min.
Gently press embryos with a glass slide.
1 min.
Remove deciliation buffer and embryos
Remove the glass slide and embryos.
Fix the coverslip in methanol and stain.
Fix the coverslip in methanol and stain
B
Chemical deciliation
Ac. tubulin
B9d1
10 μm
Mem. lost
TZ
C
Mechanical deciliation
Ac. tubulin
B9d1
10 μm
Mem. intact
TZ
D
Loss of ciliary membrane upon chemical deciliation
300 nm
300 nm
Loss of transition zone upon deciliation
E
Pre.
F
0 mins.

 © The Author(s)

Figure EV2. Transition zone is removed with cilia during deciliation.

(**A**) Schematic of the chemical and mechanical deciliation methods. (**B**) Cilia with chemical deciliation lose the B9d1 signal, possibly due to the loss of membrane with detergent in the deciliation buffer, whereas (**C**) the B9d1 signal (marked by white arrows) is maintained with mechanical deciliation. (**D**) Electron micrographs of cilia from the chemical deciliation method lack the ciliary membrane and show splaying of axonemal microtubules. (**E**) Representative TEM images of cilia pre-deciliation with intact TZ and (**F**) immediately post-deciliation (0 h), revealing the loss of TZ (arrows indicate TZ location).

© The Author(s)

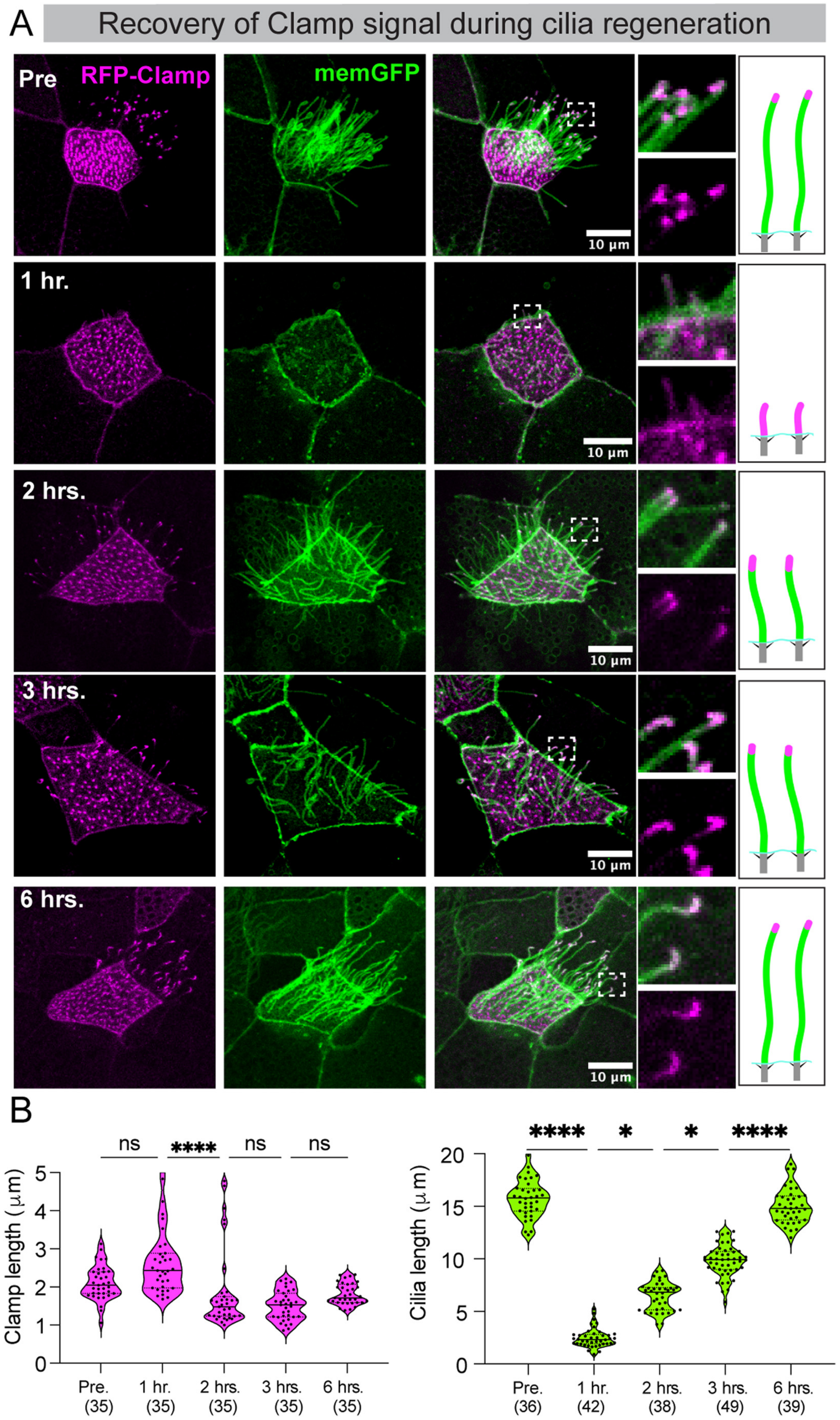
A
Recovery of Clamp signal during cilia regeneration
Pre
RFP-Clamp
memGFP
10 µm
1 hr.
10 µm
2 hrs.
10 µm
3 hrs.
10 µm
6 hrs.
10 µm
B
ns
****
ns
ns
Clamp length (µm)
0
1
2
3
4
5
Pre. (35)
1 hr. (35)
2 hrs. (35)
3 hrs. (35)
6 hrs. (35)
****
*
*
****
Cilia length (µm)
0
5
10
15
20
Pre. (36)
1 hr. (42)
2 hrs. (38)
3 hrs. (49)
6 hrs. (39)

 © The Author(s)

Figure EV3. Clamp is localized to the ciliary axoneme during the early stages of regeneration.

(**A**) MCCs are labeled with RFP-Clamp (ciliary tip and base, magenta) and memGFP (cilia, green) at various stages of cilia regeneration. After 1 h post-deciliation, the Clamp signal can be seen in the ciliary axonemes (magenta transparent). At 2 h, the Clamp signal starts accumulating at the ciliary tips. At 3 and 6 h, the Clamp signal appears more like pre-deciliated samples. (**B**) Clamp signal length (left panel) and cilia length (right panel) were measured and compared among different time points. The values in parenthesis indicate the number of cilia measured from three trials using 9–10 embryos. $^{****}P < 0.0001$; values on the comparison bar denote P value; ns - not significant, Kruskal–Wallis test, followed by Dunn's test.

© The Author(s)

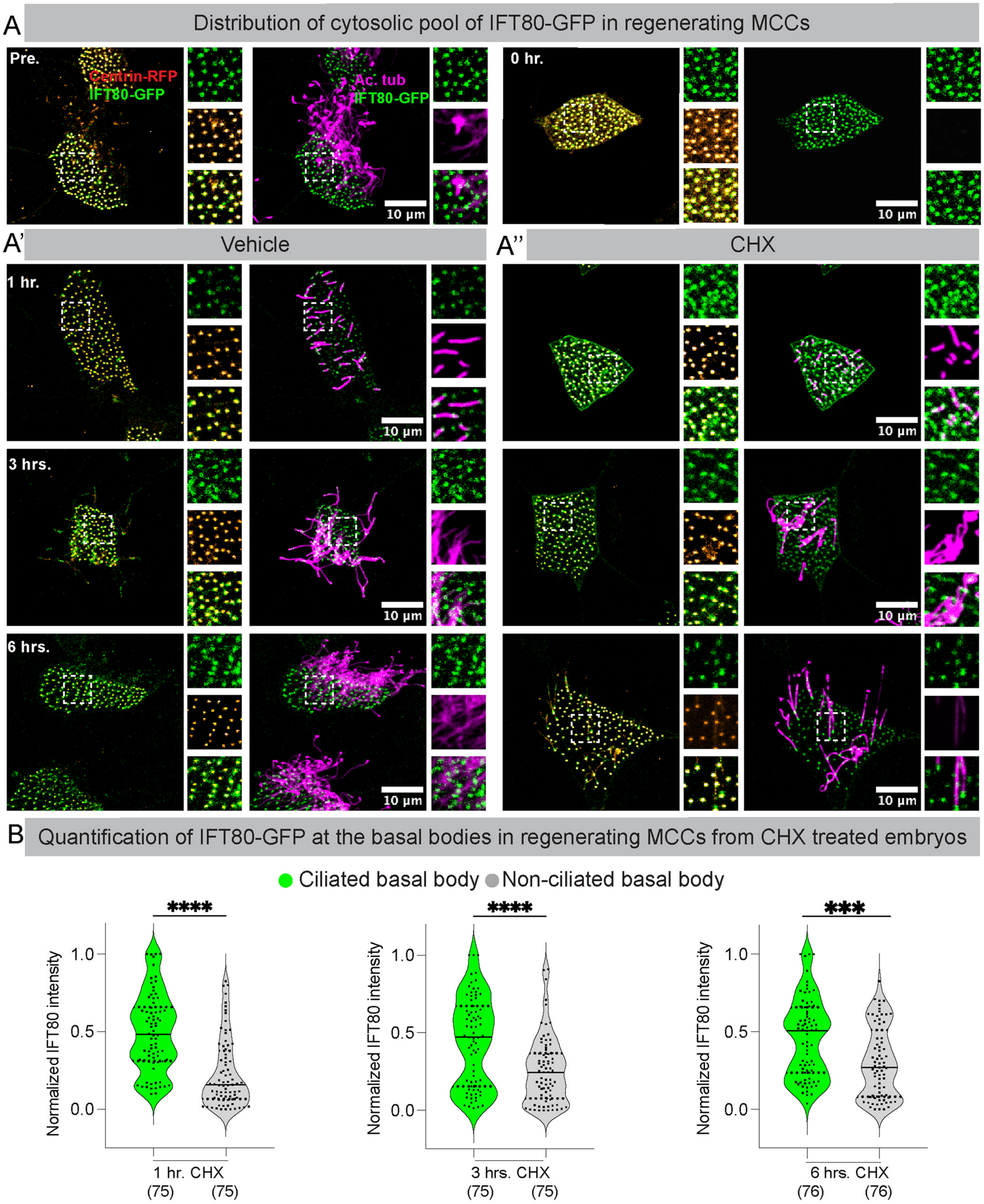
A
Distribution of cytosolic pool of IFT80-GFP in regenerating MCCs
Pre.
Centrin-RFP
IFT80-GFP
Ac. tub
IFT80-GFP
10 μm
0 hr.
10 μm
A'
Vehicle
A''
CHX
1 hr.
3 hrs.
6 hrs.
B
Quantification of IFT80-GFP at the basal bodies in regenerating MCCs from CHX treated embryos
Ciliated basal body
Non-ciliated basal body
Normalized IFT80 intensity
1.0
0.5
0.0
****
***
1 hr. CHX
(75) (75)
3 hrs. CHX
(75) (75)
6 hrs. CHX
(76) (76)

 © The Author(s)

Figure EV4. Distribution of ciliary precursor pool (IFT80-GFP) in MCCs.

(**A**) Embryos injected with IFT80-GFP (green) and Centrin-RFP (orange hot, basal bodies) were deciliated at stage 28 (0 h) and were split into two experiments (DMSO and CHX). (**A′**, **A″**) The embryos in both sets regenerated cilia for 6 h. After fixation, the embryos were stained for cilia(magenta). Note that the control MCCs at all time points (1 h, 3 h, and 6 h) have multiple cilia regenerating, and the IFT80-GFP intensity is uniform at every basal body. In contrast, the number of regenerating cilia decreases with time in CHX-treated samples, and the IFT80-GFP is enriched at a few ciliated basal bodies. (**B**) The intensity of IFT80-GFP associated with ciliated (green) vs. non-ciliated(gray) basal bodies in the same MCC (in CHX-treated samples) at different time points during cilia regeneration. A total of 8–10 basal bodies per MCC (4–5 ciliated and 4–5 non-ciliated) and 5 MCCs were chosen, and the mean gray value was estimated and normalized to the maximum and the minimum values in the set. The value in parenthesis indicates the number of basal bodies analyzed (with and without IFT80-GFP) from 9 embryos from three independent trials. Note the significant difference in the IFT80-GFP signal intensity at ciliated vs. non-ciliated basal bodies at all time points. ****$P < 0.0001$, values on the comparison bar denote *p* value; Kruskal-Wallis test, followed by Dunn's multiple comparison test.

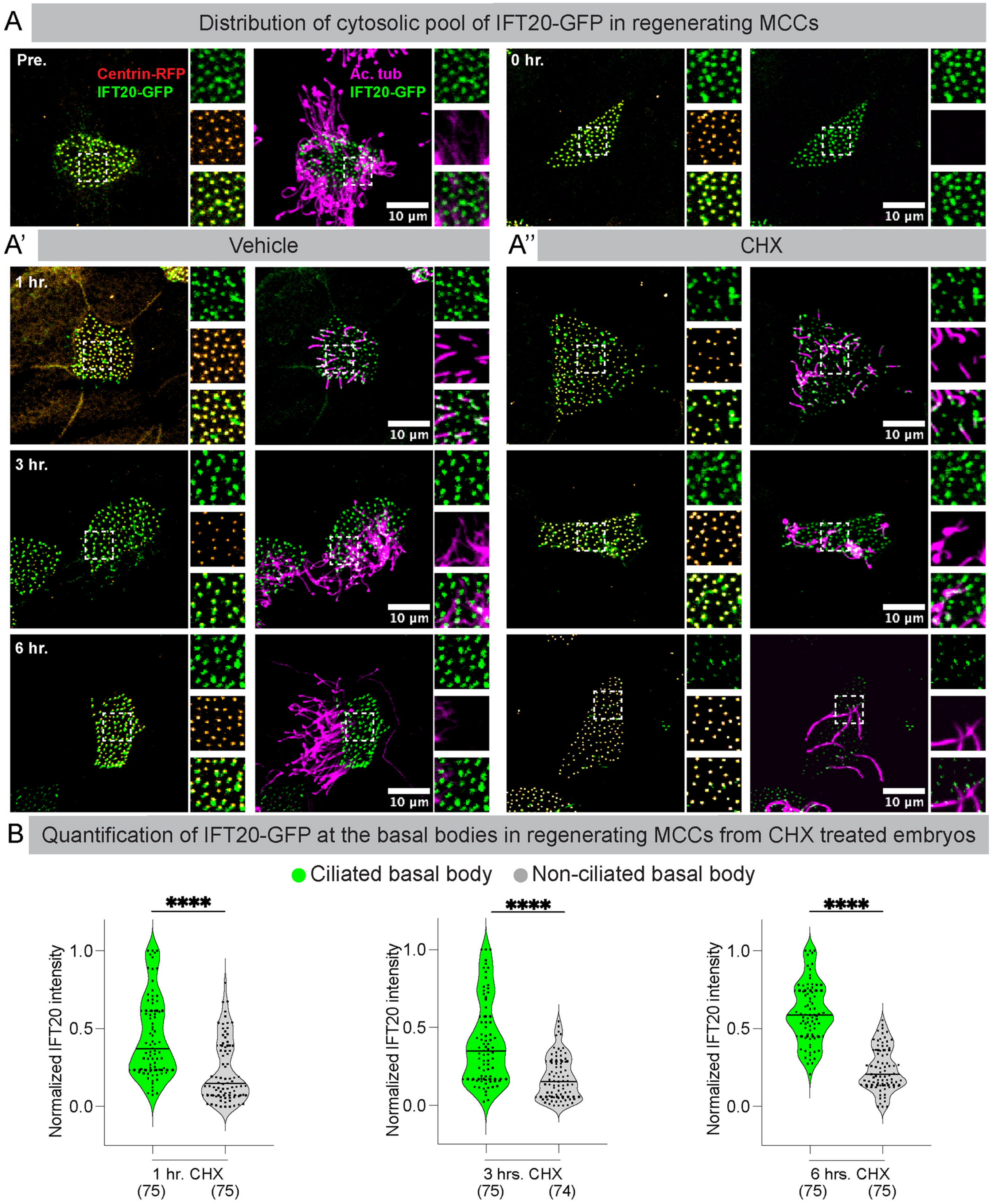
A
Distribution of cytosolic pool of IFT20-GFP in regenerating MCCs
Pre.
Centrin-RFP
IFT20-GFP
Ac. tub
IFT20-GFP
0 hr.
10 µm
A'
Vehicle
A''
CHX
1 hr.
3 hr.
6 hr.
B
Quantification of IFT20-GFP at the basal bodies in regenerating MCCs from CHX treated embryos
Ciliated basal body
Non-ciliated basal body
****
Normalized IFT20 intensity
1.0
0.5
0.0
1 hr. CHX
(75)
(75)
3 hrs. CHX
(75)
(74)
6 hrs. CHX
(75)
(75)

 © The Author(s)

◀ **Figure EV5. Distribution of ciliary precursor pool (IFT20-GFP) in MCCs.**

(**A**) Embryos injected with IFT20-GFP (green) and Centrin-RFP (orange hot, basal bodies) were deciliated at stage 28 (0 h) and were split into two experiments (DMSO and CHX). (**A′**, **A″**) The embryos in both sets regenerated cilia for 6 h. After fixation, the embryos were stained for cilia(magenta). Note that the control MCCs at all time points (1 h, 3 h, and 6 h) have multiple cilia regenerating, and the IFT20-GFP intensity is uniform at every basal body. In contrast, the number of regenerating cilia decreases with time in CHX-treated samples, and the IFT20-GFP is enriched at a few ciliated basal bodies. (**B**) The intensity of IFT20-GFP associated with ciliated (green) vs. non-ciliated(gray) basal bodies in the same MCC (in CHX-treated samples) at different time points during cilia regeneration. A total of 8–10 basal bodies per MCC (4–5 ciliated and 4–5 non-ciliated) and 5 MCCs were chosen, and the mean gray value was estimated and normalized to the maximum and the minimum values in the set. The value in parenthesis indicates the number of basal bodies analyzed (with and without IFT20-GFP) from 9 embryos from three independent trials. Note the significant difference in the IFT20-GFP signal intensity at ciliated vs. non-ciliated basal bodies at all time points. ****$P < 0.0001$, Kruskal–Wallis test, followed by Dunn's multiple comparison test.

© The Author(s)
